# Structural Insights into an Antiparallel Chair‐Type G‐Quadruplex From the Intron of *NOP56* Oncogene

**DOI:** 10.1002/advs.202406230

**Published:** 2025-03-06

**Authors:** Zhenzhen Yan, Axin He, Liqi Wan, Qian Gao, Yan Jiang, Yang Wang, Ercheng Wang, Changling Li, Yingquan Yang, Yingjie Li, Pei Guo, Da Han

**Affiliations:** ^1^ Zhejiang Cancer Hospital Hangzhou Institute of Medicine (HIM) Chinese Academy of Sciences Hangzhou Zhejiang 310022 China; ^2^ Institute of Molecular Medicine (IMM) Renji Hospital School of Medicine, Shanghai Jiao Tong University Shanghai 200127 China; ^3^ Hangzhou Institute for Advanced Study University of Chinese Academy of Sciences Hangzhou Zhejiang 310013 China; ^4^ Zhejiang Laboratory Hangzhou Zhejiang 311100 China; ^5^ College of Chemistry and Materials Science Shanghai Normal University Shanghai 200234 China; ^6^ Department of Pharmacology School of Basic Medical Sciences Peking University Health Science Center Beijing 100191 China

**Keywords:** DNA structure, G‐quadruplex, gene regulation, *NOP56* intron, solution NMR

## Abstract

G‐quadruplex (G4) structures play important roles in various biological processes, especially the gene regulation. Nucleolar protein 56 (NOP56) is an essential component in ribosome biogenesis while its overexpression associates with various types of cancers, rendering it a significant therapeutic target. Here for the first time, an antiparallel chair‐type G4 structure formed by a 21‐nt DNA sequence from the intron 1 of *NOP56* is reported, and its high‐resolution structure is determined using solution nuclear magnetic resonance spectroscopy. The *NOP56*‐G4 has a special fold containing two G‐tetrads and a C·G·C·G tetrad, which is further capped by a C∙C base pair. The G4 ligand pyridostatin (PDS) binds at the terminal G‐tetrad through π–π stacking and electrostatic interactions, increasing the melting temperature of *NOP56*‐G4 by ≈14 °C. This study further shows that PDS can significantly reduce *NOP56* mRNA levels in three cancer cell lines. This work provides an unprecedented high‐resolution structural basis for a special G4 structure from the intron of *NOP56* and suggests a feasibility of targeting intronic G4 for gene regulation, propelling new avenues for G4 structure‐based drug design and therapeutic strategy.

## Graphical abstract

This study first reports that the intron 1 of *NOP56* oncogene forms an antiparallel chair‐type G4 structure composed of two G‐tetrads and one C∙G∙C∙G tetrad. *NOP56* gene transcription can be inhibited by PDS that binds and stabilizes *NOP56*‐G4. Solution NMR structures of the free *NOP56*‐G4 and *NOP56*‐G4‐PDS complex provide valuable insights into G4 structure‐based drug design and cancer therapy.

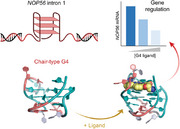

## 1 Introduction

G‐quadruplex (G4) is a type of secondary structure of nucleic acids formed by guanine‐rich sequences. Typical G4s contain three layers of G‐tetrads stabilized by metal ions (e.g., K^+^ and Na^+^) with four continuous G‐columns linked by propeller, lateral, or diagonal loops, and they are classified into parallel, hybrid, and antiparallel G4s according to the orientations of four G‐columns.^[^ ^1^ ^]^ Structural polymorphism of G4s can arise from the compositions of loops and G‐tetrads, resulting in the formation of G4 structures with various topologies, including those with only two‐layer G‐tetrads, guanine‐vacancy G‐tetrad(s), bulge, snapback loop or quadruplex‐duplex junction.^[^ ^2^ ^]^ High‐throughput sequencing has revealed over 700 000 putative G4‐forming sites in the human genome.^[^ ^3^ ^]^ G4s play important regulatory roles in various biological processes, especially the gene regulation.^[^ ^4^ ^]^ In particular, several oncogenes, including *MYC*,^[^ ^5^ ^]^ *EGFR*,^[^ ^6^ ^]^ *KRAS*,^[^ ^7^ ^]^ *c‐KIT*,^[^ ^8^ ^]^ *VEGF*,^[^ ^9^ ^]^ and *PDGFR‐β*,^[^ ^10^ ^]^ can form G4s in their promoters. Stabilizing G4 structures in these oncogene promoters by small‐molecule ligands can inhibit gene transcription, establishing G4s as promising drug targets for developing novel cancer therapeutic strategies.^[^ ^4^, ^7^, ^11^ ^]^

Nucleolar protein 56 (NOP56) is a key component of the C/D box small nucleolar ribonucleoprotein (snoRNP) complex with an essential function in ribosome biogenesis.^[^ ^12^ ^]^ Besides, NOP56 and other snoRNPs play an important role in cell transformation and tumorigenesis.^[^ ^13^ ^]^ NOP56 is found to be overexpressed in multiple types of cancers, including the colon adenocarcinoma, esophageal carcinoma, lung adenocarcinoma, and *KRAS*‐mutant cancers.^[^ ^14^ ^]^ A high level of *NOP56* expression correlates with low survival probability of *KRAS*‐mutant cancers through regulating homeostasis of reactive oxygen species, and the knockdown of *NOP56* could profoundly enhance the *KRAS*‐mutant cancer cell death.^[^ ^14b^ ^]^ These pose *NOP56* as an important target for cancer therapy. However, there have been no ligands or inhibitors that directly target the NOP56 protein, raising a high demand of developing novel strategies for *NOP56* gene regulation.

The intron 1 of wild‐type *NOP56* contains four to eight guanine‐rich GGCCTG DNA repeats.^[^ ^15^ ^]^ Although DNA sequences composed of exactly four to eight GGCCTG repeats have been reported to form hairpin and duplex structures,^[^ ^16^ ^]^ their potentials of forming G4 structures may be underestimated due to a lack of considering more subtle factors such as flanking residues. Up to now, it remains elusive if the intron 1 sequence of *NOP56* can form any intramolecular G4 structures, and if an intronic G4 can be targeted to inhibit gene transcription. In addition, the significance of studying *NOP56* intronic G4 is further elevated by a growing interest in understanding the structure and function of G4s relevant to neurodegenerative diseases,^[^ ^17^ ^]^ as an expansion of the GGCCTG repeats in *NOP56* intron 1 also associates with spinocerebellar ataxia type 36.^[^ ^15^ ^]^

In this study, we report the first solution nuclear magnetic resonance (NMR) structure of an antiparallel chair‐type G4 formed by a 21‐nt sequence from the intron 1 of wild‐type *NOP56*. The *NOP56*‐G4 adopted a special fold composed of two G‐tetrads and one C·G·C·G tetrad, and it could be bound and stabilized by pyridostatin (PDS). We determined the solution NMR structure of *NOP56*‐G4‐PDS complex and elucidated the binding mechanism. Finally, we showed that PDS could reduce *NOP56* mRNA levels in three cancer cell lines, suggesting a potential of targeting the intronic G4 to regulate *NOP56* gene transcription.

## 2 Results

### 2.1 Formation of an Antiparallel G4 Structure From the Intron 1 of *NOP56*

**Figure 1:**
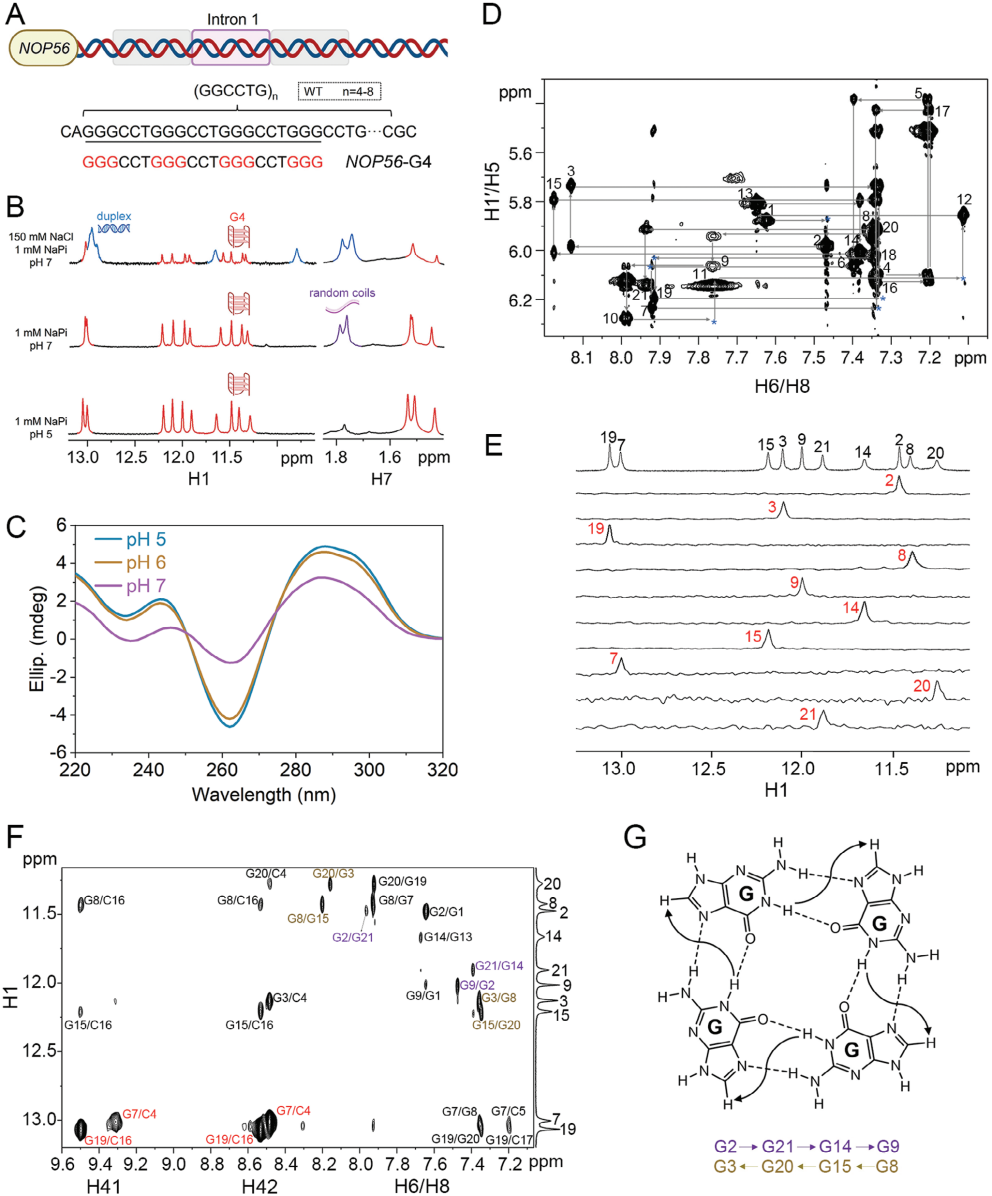
NMR and CD spectroscopic characterizations of *NOP56*‐G4. A) Schematic of the guanine‐rich GGCCTG repeats in intron 1 of the wild‐type *NOP56*. B) 1D ^1^H NMR showing guanine imino proton H1 and thymine methyl proton H7 signals of *NOP56*‐G4 in 150 mm NaCl and 1 mm NaPi at pH 7 (top), only 1 mm NaPi at pH 7 (middle), and only 1 mm NaPi at pH 5 (bottom). NMR signals from the G4, duplex, and random coils are labeled by red, blue, and purple colors, respectively. The peak intensity of G H1 was amplified by eight folds relative to T H7. C) CD spectra of *NOP56*‐G4 in 1 mm NaPi at pH 7 (purple), pH 6 (yellow), and pH 5 (blue). D) The H6/H8‐H1′ fingerprint region from NOESY spectrum of *NOP56*‐G4 in 99.96% D_2_O. The missing H6/H8‐H1′ sequential NOEs are marked with blue asterisks. (E) ^15^N‐filtered spectra of the ten 6% isotopically labeled *NOP56*‐G4 in 10% D_2_O. F) The H1‐H41/H42/H6/H8 region from NOESY spectrum of *NOP56*‐G4 in 10% D_2_O. NOEs that support the G2·G21·G14·G9, G3·G8·G15·G20, and C4·G7·C16·G19 tetrads are labeled in purple, yellow, and red colors, respectively. G) A schematic for the G‐tetrad, where the direction of arrow represents the Hoogsteen hydrogen bond donor‐to‐acceptor directionality (donor = H1/H21, and acceptor = O6/N7) which can be determined based on the internucleotide G H1‐G H8 NOE. T = 30 °C for D) and 25 °C for the else.

We first examined the G4‐forming capability of a 21‐nt d(GGGCCT)_3_GGG, which is a natural sequence pieced in the intron 1 of wild‐type *NOP56* and fulfills the theoretical criterion of forming an intramolecular G4 structure, i.e., G_3‐5_N_1‐7_G_3‐5_N_1‐7_G_3‐5_N_1‐7_G_3‐5_ (N = A, G, C or T).^[^ ^1b^ ^]^ Herein we named it as *NOP56*‐G4 (**Figure** **1**A; Table S1, Supporting Information). We acquired the 1D ^1^H NMR spectrum for *NOP56*‐G4 in a sodium phosphate (NaPi) buffer at pH 7 with 150 mm NaCl, a physiologically relevant ionic condition. The imino proton (G H1 and T H3) region showed two sets of NMR signals from two DNA conformers, respectively, including one set of G H1 signals at ≈11.3 to 12.2 ppm arising from Hoogsteen base pairs in G‐tetrads of G4 structure, and another set of G H1 signals at ≈13.0 ppm arising from Watson‐Crick base pairs and T H3/G H1 signals at ≈11.7/10.8 ppm arising from T∙G mismatches^[^ ^16^ ^]^ of duplex structure (Figure 1B, top). The atomic numbering of deoxyribonucleotide is shown in Figure S1 (Supporting Information). The coexistence of duplex structure in 150 mm Na^+^ was also supported by its similar electrophoretic mobility to a 21‐bp duplex reference in the native polyacrylamide gel electrophoresis (PAGE) assay (Figure S2A, Supporting Information). This conformational heterogeneity hindered further NMR structural characterization of the G4 structure.

To promote the population of G4 structure, we depleted NaCl because Na^+^ was known to stabilize duplex structure by neutralizing the negatively charged phosphodiester backbones of inter‐chains.^[^ ^18^ ^]^ Depleting NaCl successfully vanished the duplex structure (Figure S2A,B, Supporting Information), but led to the formation of a third conformer that showed T H7 signals at ≈1.8 ppm (Figure 1B, middle). We further found that upon lowering the pH (1 mm NaPi at pH 5), T H7 signals from the third conformer vanished and only one set of G4 signals was observed (Figure 1B, bottom). As for the third conformer appeared in 1 mm NaPi at pH 7 (Figure 1B, middle), it might arise from an alternative loop conformation of G4 or random coils. If the T H7 signals at ≈1.8 ppm were from another loop conformation of G4, two sets of H1 signals would be observed at pH 7, or the H1 signal intensity would be similar at pH 7 and pH 5. However, only one set of H1 signals was observed at pH 7 and pH 5, and the overlaid NMR spectra showed that the H1 signals at pH 5 were much more intense than those at pH 7 (Figure S2C, Supporting Information). These suggest that the third conformer was random coils. NMR features of the G4 structure include eight well‐resolved G H1 signals at ≈11.3 to 12.2 ppm and two G H1 signals at ≈13.0 ppm, suggesting that it contained two G‐tetrad layers and two C‐G Watson‐Crick base pairs. We further showed that this G4 still formed at physiologically relevant K^+^ concentrations as suggested by the observation of G H1 signals at ≈11.3‐12.2 ppm and ≈13.0 ppm in 50/70/150 mm K^+^, although its population decreased due to the competitive formation of other conformer(s) (Figure S3, Supporting Information).

The circular dichroism (CD) spectra of *NOP56*‐G4 showed two positive bands at ≈247/290 nm and a negative band at 262 nm, indicating an antiparallel G4 topology (Figure 1C). Consistent with the NMR data shown in Figure 1B, CD features of the antiparallel G4 were more obvious at pH 5 than pH 7. We further determined the melting temperature (*T_m_*) of *NOP56*‐G4 by measuring the CD intensity at 295 nm as a function of temperature, and found that the *T_m_* of *NOP56*‐G4 at pH 5 was higher by ≈2 °C than that at pH 7 (Figure S4, Supporting Information). The pH‐dependent stability of *NOP56*‐G4 is similar to some G4s with a relatively high cytosine content^[^ ^19^ ^]^ and i‐motif structures that usually gain thermostability at acidic pH.^[^ ^20^ ^]^ We then proceeded to elucidate the unusual structure of *NOP56*‐G4 by 2D NMR.

### 2.2 Solution NMR Structure of the Antiparallel Chair‐Type *NOP56*‐G4

As the 1D and 2D NMR spectral features of the G4 structure at pH 7 and pH 5 were generally similar (Figure 1B; Figure S5, Supporting Information), we determined the solution NMR structure of *NOP56*‐G4 at pH 5 owing to the higher spectral quality. The 2D nuclear Overhauser effect spectroscopy (NOESY) H6/H8‐H1′ fingerprint region of *NOP56*‐G4 is shown in Figure 1D. The C4 H41/H42 (9.31/8.49 ppm) and C16 H41/H42 (9.50/8.53 ppm) were assigned based on the intranucleotide cytosine H5‐H41/H42 NOEs (Figure S6, Supporting Information), which were similar NMR features to those of cytosine residues involved in the C∙G∙C∙G tetrad.^[^ ^21^ ^]^ Together with the eight G H1 signals at ≈11.3‐12.2 ppm implicating two G‐tetrads, we suspected that G2/G3 in the first G‐tract and G14/G15 in the third G‐tract constituted two G‐columns, respectively. Accordingly, we prepared ten *NOP56*‐G4 samples with a 6% ^13^C/^15^N isotopic labeling at the designated position of G2, G3, G7, G8, G9, G14, G15, G19, G20 and G21, respectively, and assigned their H1 and H8 resonances using ^1^H‐^15^N heteronuclear single quantum coherence (HSQC) and ^1^H‐^13^C HSQC spectra, respectively (Figure 1E; Figure S7, Supporting Information). The other proton resonances including C H5, T H7, and sugar protons were assigned from 2D double‐quantum filtered correlation spectroscopy (DQF‐COSY) and total correlation spectroscopy (TOCSY) spectra (Figures S8–S10 and Table S2, Supporting Information).

**Figure 2:**
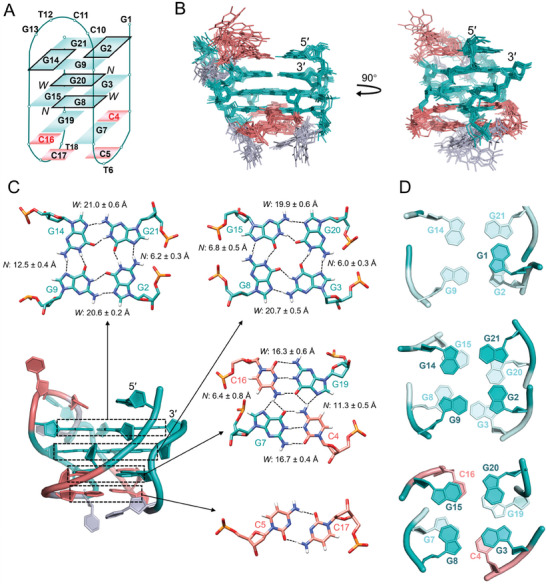
Solution NMR structures of the free *NOP56*‐G4. A) Schematic showing the secondary structure of *NOP56*‐G4. Guanine and cytosine residues are shown in green and red colors, respectively. Guanine residues (G2, G8, G14, and G20) with a *syn* glycosidic torsion are highlighted by a black outline. N and W represent narrow and wide grooves, respectively. B) The 10 superimposed solution NMR structures of free *NOP56*‐G4 (PDB ID: 8XGP). C) One structure with the lowest total energy shows details of the two G‐tetrads, C4·G7·C16·G19 tetrad and C5·C17 base pair. The widths of narrow and wide grooves are represented as mean ± S.D. from the 10 representative structures. D) Stackings between G1 and G2·G21·G14·G9 tetrad (top), between G2·G21·G14·G9 and G3·G8·G15·G20 tetrads (middle), and between G3·G8·G15·G20 and C4·G7·C16·G19 tetrads (bottom).

The G H1 assignments shown in Figure 1E suggest that G2, G3, G8, G9, G14, G15, G20 and G21 formed two G‐tetrads. We determined the Hoogsteen hydrogen bond donor‐to‐acceptor directionality (indicated by the direction of arrow) of each G‐tetrad to be G2→G21→G14→G9 and G3←G20←G15←G8, based on i) NOEs of G2 H1‐G21 H8, G21 H1‐G14 H8 and G9 H1‐G2 H8, and ii) NOEs of G3 H1‐G8 H8, G8 H1‐G15 H8, G15 H1‐G20 H8 and G20 H1‐G3 H8, respectively (Figure 1F,G). Besides, the upfield C8 signals of G3/G7/G9/G15/G19/G21 at ≈138 ppm and the downfield C8 signals of G2/G8/G14/G20 at ≈142 ppm suggested their *anti* and *syn* glycosidic torsion angles, respectively (Figure S7, Supporting Information).^[^ ^22^ ^]^ In addition, the formation of C4∙G7∙C16∙G19 tetrad is supported by several lines of evidence. First, C4‐G7 and C16‐G19 adopted Watson‐Crick base pairs as evidenced by NOEs of C4 H41/H42‐G7 H1 and C16 H41/H42‐G19 H1 (Figure 1F). The amino protons of H41/H42 of C4 and C16 resonated at 9.31/8.49 and 9.50/8.53 ppm, respectively (Figure 1F; Table S2, Supporting Information), which were similar to those in the reported C∙G∙C∙G tetrads (9.15‐9.23/8.49‐8.88 ppm).^[^ ^21^, ^23^ ^]^ These cytosine amino H41/H42 signals were much downfield than those in regular Watson‐Crick base pairs (8.6‐8.0/7.0‐6.3 ppm)^[^ ^24^ ^]^ due to the more pronounced deshielding effects.^[^ ^21^, ^23^ ^]^ Second, the NOEs of G7 H8‐C16 H42/H5/H6 and G19 H8‐C4 H42/H5/H6 squared with the formation of a C∙G∙C∙G tetrad with direct alignment where the distances between guanine H8 from one Watson‐Crick base pair and cytosine H42/H5/H6 from the other Watson‐Crick base pair are within 6 Å (Figure S11A,B, Supporting Information).^[^ ^21^, ^25^ ^]^ Third, the NOEs of C4 H42‐G3/G20 H1, G7 H1‐G3 H1, C16 H42‐G8/G15 H1 and G19 H1‐G15 H1 revealed that all residues in the C4∙G7∙C16∙G19 tetrad stacked on the neighboring G3·G8·G15·G20 G‐tetrad, further consolidating the co‐planarity of C4, G7, C16 and G19 and thus the C4∙G7∙C16∙G19 tetrad (Figure S11C, Supporting Information). Taken together, the G2·G21·G14·G9, G3·G8·G15·G20 and C4∙G7∙C16∙G19 tetrads delineated a secondary structure shown in **Figure** **2**A.

We evaluated the relative structural stability of the two G‐tetrads and C4·G7·C16·G19 tetrad by deuterium oxide (D_2_O) exchange NMR experiments. Immediately after dissolving *NOP56*‐G4 in 99.96% D_2_O, G7 and G19 H1 signals from the C4∙G7∙C16∙G19 tetrad vanished, whereas G2/G9/G21 H1 signals from the G2·G21·G14·G9 tetrad and G3/G8/G15/G20 H1 signals from the G3·G8·G15·G20 tetrad remained observable (Figure S12, Supporting Information), revealing a relatively higher structural stability of the two G‐tetrads than C·G·C·G tetrad in *NOP56*‐G4. We noticed that for C5 and C17, their H41 and H42 signals were downfield at 8.6 and 7.6 ppm, respectively (Figure S13A, Supporting Information), suggesting that the amino groups of C5 and C17 formed base pairing. The H41/H42 chemical shifts of C5 and C17 are also similar to those of the C·C mismatch in the G4 structure formed by a mutant of *PDGFR‐β* promoter.^[^ ^26^ ^]^ Although the pH‐dependent thermostability of *NOP56*‐G4 hinted a possibility for semi‐protonation of C·C, the NMR spectra of *NOP56*‐G4 did not show any protonated cytosine H3 signal at ≈15.0 to 17.0 ppm at pH 5/6/7 (Figure S13B, Supporting Information) probably due to a fast exchange with solvent. To avoid overestimations of C5·C17 base pairing and semi‐protonation of C5·C17, we used non‐protonated C5 and C17 without adding any base pairing restraints for structural calculations.

The solution NMR structure of *NOP56*‐G4 was calculated using restrained molecular dynamics (rMD) simulations based on 313 NOE‐derived distance restraints and other structural restraints (Tables S3–S7, Supporting Information). Ten structures with the lowest total energies were selected as the final representative ensemble (Figure 2B) and their NMR refinement statistics are shown in **Table** **1**. The core of *NOP56*‐G4 comprised of G2·G21·G14·G9, G3·G8·G15·G20, and C4∙G7∙C16∙G19 tetrads was well defined with a small heavy‐atom root‐mean‐square deviation (RMSD) of 0.5 ± 0.1 Å. There were three lateral loops, including C5‐T6, C17‐T18 and C10‐C11‐T12‐G13. For a clearer representation, the structure with the lowest total energy was used to show detailed structural features (Figure 2C).

**Table 1:** NMR restraints and refinement statistics of the free *NOP56*‐G4 and *NOP56*‐G4‐PDS complex.

	*NOP56*‐G4	*NOP56*‐G4‐PDS
*Structural restraints*		
NOE‐derived distance	313	290
Intra‐residue	197	209
Inter‐residue	116	81
G4‐ligand	/	8
Hydrogen bond distance	10	10
Glycosidic torsion angle (χ)	18	19
G‐tetrad planarity	24	24
Chirality	63	63
*Restraint deviations*		
No. of distance deviation >0.2 Å	0	0
Max. distance deviation (Å)	0.06	0.031
No. of angle deviation >5°	0	0
Max. angle deviation (°)	2.19	1.25
*Deviations from ideal geometry*		
Deviation from ideal bond (Å) ^a)^	0.0096 ± 0.0003	0.0099 ± 0.0003
Deviation from ideal angle (°) ^a)^	2.51 ± 0.06	2.61 ± 0.05
*Heavy‐atom RMSD from mean structure (Å)*		
All residues ^a^ ^)^	0.91 ± 0.09	0.7 ± 0.1
G‐tetrads and C·G·C·G tetrad ^a)^	0.5 ± 0.1	0.47 ± 0.09

^a)^ Data are represented as mean ± S.D. from the 10 representative solution NMR structures.

**Figure 3:**
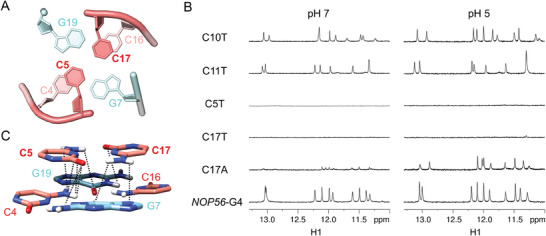
Structural importance of the stacking between C4·G7·C16·G19 and C5∙C17. A) Stacking between C4·G7·C16·G19 and C5∙C17 in *NOP56*‐G4. B) 1D ^1^H NMR spectra (G H1 region) of C10T, C11T, C5T, C17T, C17A and *NOP56*‐G4 at pH 7 and pH 5. [DNA] = 0.2 mm, [NaPi] = 1 mm, 25 °C. C) Electrostatic interactions between C4·G7·C16·G19 and C5∙C17. Favorable electrostatic interactions with atom‐atom distances within 4 Å are represented by black dotted lines.

The G2·G21·G14·G9 and G3·G8·G15·G20 G‐tetrads exhibited extensive base‐base stackings, with the latter being further stacked with C4·G7·C16·G19 tetrad (Figure 2D). In the C4·G7·C16·G19 tetrad, C4‐G7 and C16‐G19 adopted Watson‐Crick base pairs. The H42 protons of C4 and C16 formed bifurcated hydrogen bonds with G19 O6/N7 and G7 O6/N7, respectively. C5 and C17 capped on the C4·G7·C16·G19 tetrad and predominantly formed a two‐hydrogen‐bond base pair in the calculated structures (eight out of ten) (**Figure** **3**A; Figure S14, Supporting Information). The symmetric carbonyl‐amino pairing geometry of C5∙C17 in *NOP56*‐G4 is similar to the C·C in the G4 structure adopted by a mutant of *PDGFR‐β* promoter,^[^ ^26^ ^]^ and both of C·C base pairs had similarly downfield H41/H42 chemical shifts. To test if the C5∙C17 contributed to the stabilization of *NOP56*‐G4, we first designed four sequences by replacing C5, C10, C11, and C17 with a thymine, denoted as C5T, C10T, C11T, and C17T, respectively. C10T and C11T adopted G4s as suggested by their G H1 signals similar to those of *NOP56*‐G4, while C5T and C17T exhibited much weaker G H1 signals (Figure 3B). We further substituted C17 with an adenine (C17A), which showed weaker G H1 signals than *NOP56*‐G4 at both pH 5 and 7. The G H1 signals of C17A at pH 5 were more intense than those of C17A at pH 7, which might be attributed to the protonation of C∙A base pair^[^ ^27^ ^]^ that stabilized the G4 structure. In addition, we identified electrostatic interactions between C4·G7·C16·G19 and C5∙C17 (Figure 3C). Overall, both C5 and C17 were important to stabilize the *NOP56*‐G4.

Apart from the tetrads, it was noted that the 2‐nt C5‐T6 and C17‐T18 loops connected two wide grooves, respectively, while the 4‐nt C10‐C11‐T12‐G13 loop bridged a narrow groove (Figure 2A). This can be rationalized by the capping between C4∙G7∙C16∙G19 and C5·C17. In the C4∙G7∙C16∙G19 tetrad, C4‐G7 and C16‐G19 formed Watson‐Crick base pairs across the two wide grooves with groove widths of ≈16.7 and 16.3 Å, respectively (Figure 2C), which accommodated C5‐T6 and C17‐T18 lateral loops to facilitate substantial stackings and electrostatic interactions between C4∙G7∙C16∙G19 and C5·C17 (Figure 3A,C). The two narrow grooves, especially the one with a groove width of ≈6.4 Å, appeared to be spatially unfavorable for forming C5·C17 base pair and such capping interactions. It has also been reported that a 4‐nt loop bridged a narrow groove of G‐tetrad benefitting from the loop flexibility.^[^ ^28^ ^]^ Finally, we validated the stability of the determined *NOP56*‐G4 structure by unrestrained MD simulations following the established protocol.^[^ ^6^, ^29^ ^]^ Three structures of *NOP56*‐G4 with the lowest total energies were solvated in TIP3P water model, neutralized with Na^+^ cations, and then subjected to unrestrained MD simulations for 500 ns for each structure. While the Na^+^ cannot be directly observed by NMR experiments, we followed the routine to add a Na^+^ between the two G‐tetrads for MD simulations of the solution NMR structures, as a metal ion is required to mitigate the repulsive interactions between the facing guanine O6 atoms.^[^ ^6^ ^]^ The *NOP56*‐G4 structures well persisted without significant structural rearrangements (Figure S15, Supporting Information), suggesting that the determined *NOP56*‐G4 structure is robust.

### 2.3 PDS Bound and Substantially Stabilized *NOP56*‐G4

**Figure 4:**
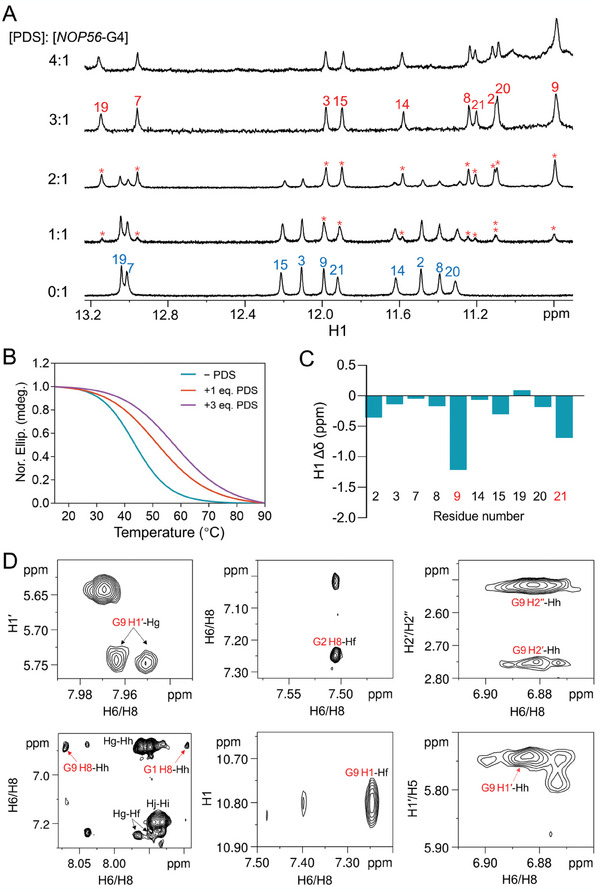
NMR and CD characterization of PDS binding to *NOP56*‐G4. A) 1D ^1^H NMR spectra of *NOP56*‐G4 upon PDS titration. Guanine H1 signals from the *NOP56*‐G4‐PDS complex are marked by red asterisks, and those from the free *NOP56*‐G4 are labeled in blue. B) Normalized CD melting curves of *NOP56*‐G4 with various concentrations of PDS by recording the CD intensity at 295 nm. The *T_m_* values of *NOP56*‐G4 were determined to be 44.8 ± 0.7, 52.4 ± 0.3 and 58.8 ± 0.2 °C at 0, 1 and 3 equivalent(s) of PDS, respectively. [NaPi, pH 7] = 1 mm. Data are represented as mean ± S.D. by three replicative experiments. C) Guanine H1 chemical shift change of *NOP56*‐G4 post binding to PDS (Δδ = δ_complex_ − δ_free_). The symbol of δ represents chemical shift. The G9 and G21 residues that showed large Δδ of H1 are highlighted in red. D) Intermolecular NOEs between *NOP56*‐G4 and PDS in the binding complex. Atoms from DNA are labeled in red. [DNA] = 0.5 mm, [PDS] = 1.5 mm, [NaPi, pH 7] = 1 mm, 25 °C.

Building upon the structural findings of *NOP56*‐G4, we wondered if *NOP56*‐G4 could be a target for small‐molecule ligands to inhibit *NOP56* gene transcription. PDS is a potent G4 ligand with high affinity to many G4s. Although it lacks binding specificity to a particular G4, it is thought to constitute a suitable model for such a proof‐of‐concept study.^[^ ^30^ ^]^ For instance, R. Rodriguez et al used PDS to reduce *SRC* proto‐oncogene expression in human breast cancer cells and validated *SRC*‐G4 as a druggable target.^[^ ^31^ ^]^ In addition, we also aimed to elucidate the binding mechanism as an effort to guide the design of better ligands for this special G4 structure in the future work. For these aims, we first acquired 1D ^1^H NMR spectra to investigate the binding of PDS to *NOP56*‐G4 at pH 7 by monitoring the G H1 and T H7 regions. At 3 equivalents of PDS, NMR signals from the free *NOP56*‐G4 and random coils completely disappeared, and only a new set of signals from the *NOP56*‐G4‐PDS complex were observed (**Figure** **4**A; Figure S16, Supporting Information, red asterisks). The signals of binding complex were not much affected upon further adding PDS to 4 equivalents. We also verified that adding a small volume of deuterated DMSO, which was used to dissolve PDS, did not affect the NMR spectra of *NOP56*‐G4 (Figure S17, Supporting Information). The CD spectra revealed that *NOP56*‐G4 in the binding complex retained an antiparallel topology (Figure S18, Supporting Information). The *T_m_* of *NOP56*‐G4 was increased by ≈14 °C at 3 equivalents of PDS as determined by recording the CD intensity at 295 nm as a function of temperature (Figure 4B).

We then determined the *NOP56*‐G4‐PDS binding complex structure at 3 equivalents of PDS in 1 mm NaPi at pH 7, owing to the high quality of NMR spectra. The NMR assignments of *NOP56*‐G4 in the complex are shown in Figures S19–S21 and Table S8 (Supporting Information), and the NMR assignments of PDS in the free and complex states are shown in Figures S22 and S23 (Supporting Information). The large chemical shift changes (Δδ) of G9 H1 and G21 H1 posted binding to PDS (Figure 4C) and the intermolecular NOEs between G1/G2/G9 and PDS (Figure 4D) suggested that one molecule of PDS bound at the terminal G2·G21·G14·G9 tetrad. The isothermal titration calorimetry (ITC) experiment further revealed that PDS bound to *NOP56*‐G4 at nearly a 1:1 stoichiometry with a dissociation constant of 0.64 ± 0.02 µM (Figure S24, Supporting Information). In NMR experiments, a ligand‐to‐DNA ratio higher than its binding stoichiometry is often needed to push the binding equilibrium toward the formation of a well‐resolved complex at the NMR time scale.^[^ ^7^, ^10^, ^32^ ^]^

### 2.4 Solution NMR Structures of *NOP56*‐G4‐PDS Complex

**Figure 5:**
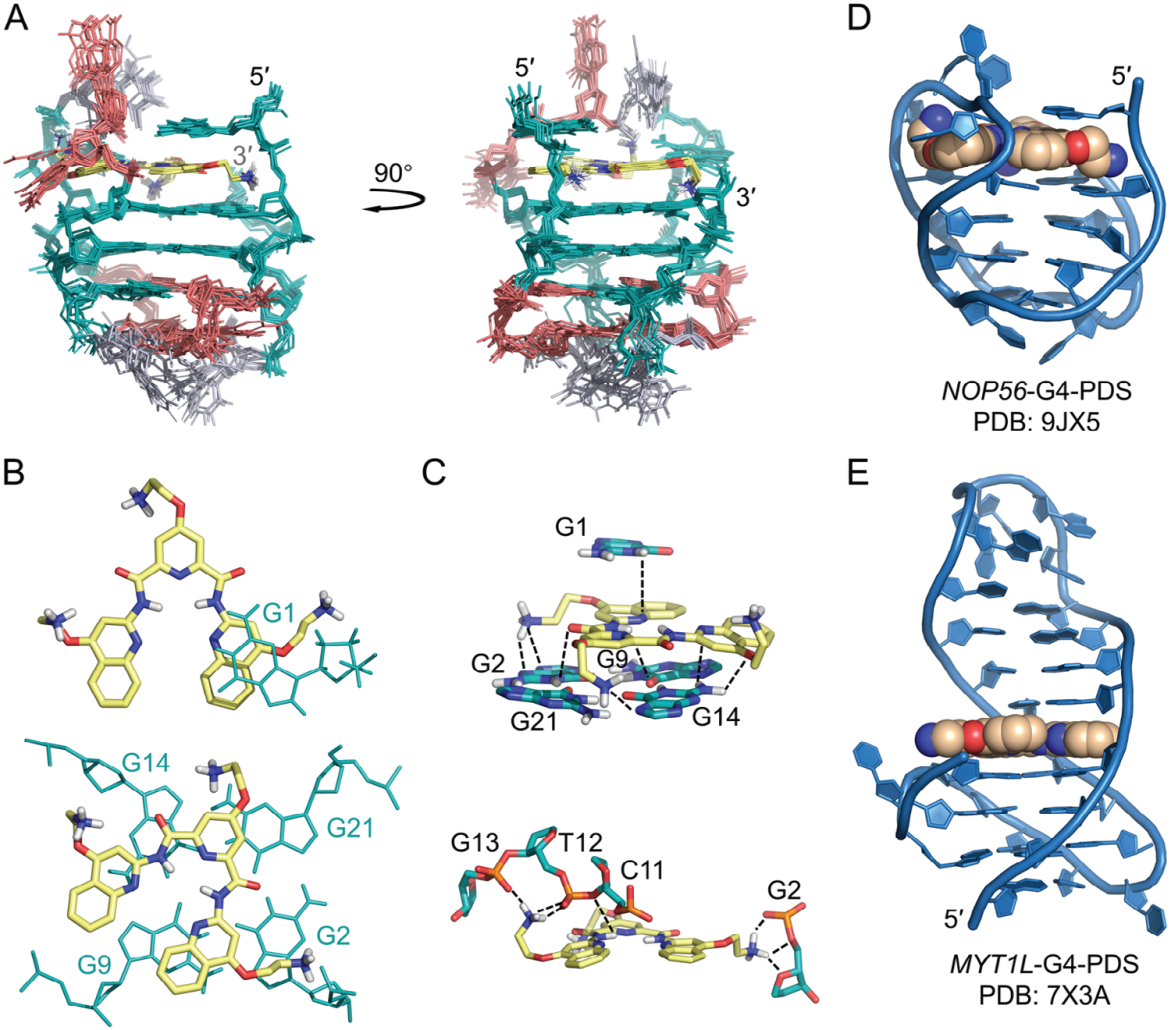
Solution NMR structures of the *NOP56*‐G4‐PDS complex. A) Superimposed ten representative solution NMR structures of the 1:1 *NOP56*‐G4‐PDS complex (PDB ID: 9JX5). B) Stick representation shows PDS (yellow) stacked between G1 and G2·G21·G14·G9 tetrad (green). C) Electrostatic interactions between PDS and the nucleobases of G1/G2/G9/G14, and between PDS and the phosphodiester backbone of G2/C11/T12/G13. Black dotted lines represent electrostatic interactions with an atom‐atom distance within 4 Å. D,E) Comparison of the G4‐ligand binding complex structures of *NOP56*‐G4‐PDS (PDB ID: 9JX5, this work) and *MYT1L*‐G4‐PDS (PDB ID: 7X3A).^[^ ^30c^ ^]^

We calculated the solution NMR structure of the *NOP56*‐G4‐PDS complex using rMD simulations with 290 NOE‐derived distance and other structural restraints (Tables S9–S13, Supporting Information). Ten structures with the lowest total energies were selected as the final representative ensemble (**Figure** **5**A) and their NMR refinement statistics are shown in Table 1. PDS bound at the terminal G2·G21·G14·G9 tetrad with its central pyridine group stacking with G14 and G21 and one of the quinoline groups stacking on G2 and G9 (Figure 5B), which well agreed with the upfield shifted H1 signals of G9 and G21 post binding (Figure 4C). Apart from stackings, there were favorable electrostatic interactions between PDS and the nucleobases of G1, G2, G9, and G14, and between PDS and the phosphodiester backbone of G2, C11, T12, and G13 (Figure 5C). Compared to the free *NOP56*‐G4 where C11 predominantly stacked on the terminal G2·G21·G14·G9 tetrad and C10 extruded out the G‐core, the *NOP56*‐G4 complexed with PDS had both C10 and C11 extruding out the G‐core (Figure S25, Supporting Information).

It is worth comparing the *NOP56*‐G4‐PDS complex with the reported *MYT1L*‐G4‐PDS complex (PDB ID: 7X3A),^[^ ^30c^ ^]^ as these two G4s adopted antiparallel and hybrid topologies with non‐canonical structural features, respectively (Figure 5D,E). In the *MYT1L*‐G4‐PDS complex, PDS bound at the quadruplex‐duplex junction using its central pyridine and two quinoline arms to form extensive stacking interactions, with PDS sandwiched between the 3′ G‐tetrad and an A‐T base pair. In the *NOP56*‐G4‐PDS complex, PDS bound at the terminal G2·G21·G14·G9 tetrad predominantly using its pyridine and one of the quinoline arms to form stackings, and the other arm was involved in electrostatic interactions with the 4‐nt C10‐C11‐T12‐G13 loop.

We further verified that the *NOP56*‐G4‐PDS complex could form at physiologically relevant K^+^ concentrations by CD spectroscopy and DNA polymerase stop assay. The CD spectrum of *NOP56*‐G4‐PDS showed CD features of the antiparallel G4, including 245 nm (+), 263 nm (‐), and 295 nm (+), in 100 mm K^+^ (Figure S26, Supporting Information). For DNA polymerase stop assay using the Klenow fragment (KF) in 100 mm K^+^, the primer extension was inhibited in a PDS concentration‐dependent manner when the *NOP56*‐G4 sequence was present in the template strand, but not affected by PDS when a 21‐nt non‐G4 sequence template was present in the template (Figure S27, Supporting Information). This suggests that PDS bound and stabilized *NOP56*‐G4 that impeded KF processivity. Collectively, our results demonstrated that the *NOP56*‐G4 could form within a more extensive DNA context, and the PDS could bind and stabilize *NOP56*‐G4 in K^+^.

### 2.5 PDS Reduced *NOP56* mRNA Levels in Cancer Cells

**Figure 6:**
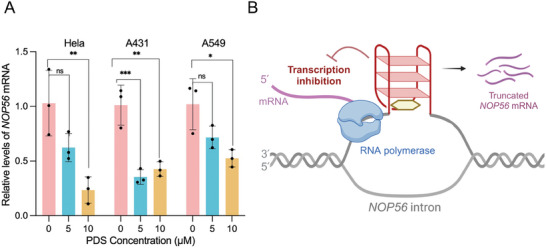
PDS inhibited *NOP56* gene transcription in cancer cells. A) RT‐qPCR results show the relative *NOP56* mRNA level in Hela, A431, and A549 cell lines with the treatment of 0, 5, or 10 µm PDS. The control group was treated with <0.1% DMSO. Data are represented as mean ± SEM by three independent experiments. Statistical analysis was performed using one‐way ANOVA followed by post hoc Dunnett test (^*^ *p* <0.05, ^**^ *p* <0.01, ^***^ *p* <0.001, ns: not significant). B) A proposed mechanism for inhibiting *NOP56* gene transcription by using small‐molecule ligand (yellow) to bind and stabilize the *NOP56* intronic G4.

Based on the above well‐established structural findings, we wondered if using small‐molecule ligands to stabilize this intronic G4 would inhibit *NOP56* gene transcription. As a proof‐of‐concept, we used PDS to treat three cancer cell lines, including the Hela, A431, and A549, and quantified *NOP56* mRNA levels using real‐time quantitative polymerase chain reaction (RT‐qPCR) assays. Although PDS can bind to many G4s without selectivity, it is thought to constitute a suitable model for such a proof‐of‐concept study,^[^ ^30^ ^]^ as exemplified by the use of PDS to reduce *SRC* proto‐oncogene expression in human breast cancer cells and to validated *SRC*‐G4 as a druggable target.^[^ ^31^ ^]^ We first measured the half‐maximal inhibitory concentration (IC_50_) values of PDS to Hela, A431, and A549 cells to be 109.2, 204.4, and 203.5 µm, respectively (Figure S28A, Supporting Information). Accordingly, PDS concentrations far below these IC_50_ values were used to examine the effect on *NOP56* gene transcription. Cells were incubated with 5 or 10 µM PDS for 24 h, and then the *NOP56* mRNA levels were measured with the *β‐actin* mRNA level serving as an internal reference. It shows that treatment with 5 or 10 µM PDS for 24 h did not affect the *β‐actin* mRNA levels in all cell lines (Figure S28B, Supporting Information). For Hela and A549 cells, a significant reduction in *NOP56* mRNA level was achieved by treatment with 10 µM PDS (**Figure** **6**A). A lower dose of 5 µM PDS could significantly reduce *NOP56* mRNA level in A431 cells. The sequences of forward and reverse primers for *NOP56* and *β‐actin* used in RT‐qPCR experiments are shown in Table S1 (Supporting Information).

## 3 Discussion and Conclusion

The *NOP56* gene is upregulated in various cancers, particularly in the *KRAS*‐mutant cancers, where its high expression is associated with poor prognosis.^[^ ^14^ ^]^ For instance, in the *KRAS*‐mutant lung cancer, the upregulation of *NOP56* is linked to the metabolic dependence of tumor cells, especially through the regulation of reactive oxygen species homeostasis. The loss of *NOP56* increased the sensitivity of tumor cells to synthetic lethality with mammalian target of rapamycin inhibitors.^[^ ^14b^ ^]^ Therefore, downregulation of *NOP56* provides a potential therapeutic strategy for the treatment of *KRAS*‐mutant cancers. A guanine‐rich repetitive sequence has been found in the intron 1 of *NOP56* (Figure 1A).^[^ ^15^ ^]^ For the first time, we report the 21‐nt d(GGGCCT)_3_GGG sequence from the intron 1 of *NOP56* can form a non‐canonical G4 structure and determine its high‐resolution structure. Comparing to the commonly observed G4s that contain two to three G‐tetrads,^[^ ^33^ ^]^ *NOP56*‐G4 adopts a special fold comprised of two G‐tetrads and one C∙G∙C∙G tetrad, which is further capped by a C∙C base pair. The capping interaction between C·G·C·G tetrad and C∙C base pair is observed in i‐motifs^[^ ^34^ ^]^ but rarely found in G4. We demonstrated that the C∙C base pair itself and its electrostatic interactions with C·G·C·G tetrad play a critical role in stabilizing the *NOP56*‐G4 (Figure 3).

Many oncogenes are found to form G4s in their promoters, such as the *MYC*, *EGFR*, *KRAS*, *c‐KIT*, *VEGF*, and *PDGFR‐β*.^[^ ^5^, ^6^, ^7^, ^8^, ^9^, ^10^ ^]^ It has been well documented that the formation of G4 structures in these oncogene promoters can either activate^[^ ^35^ ^]^ or inhibit^[^ ^31^ ^]^ transcription, depending on whether the G4 recruits or prevents association of transcription factors to the promoter. However, the roles of G4s formed in non‐promoter regions, such as the intron, in regulating oncogene expression remain much less well understood. We validated the binding of PDS to *NOP56*‐G4 and characterized the *NOP56*‐G4‐PDS complex structure (Figures 4 and 5), and demonstrated that PDS could bind and stabilize *NOP56*‐G4 in K^+^ by CD and DNA polymerase stop assay (Figures S26 and S27, Supporting Information). While the working solution for KF polymerase contained 50 mm NaCl at pH 7.9 that would be unfavorable, to some extent, for the formation of *NOP56*‐G4, the PDS showed a potency of stabilizing the *NOP56*‐G4. This agreed with the result that PDS increased the *T_m_* of *NOP56*‐G4 by ≈14 °C (Figure 4B), and the stabilizing effect of PDS on some G4s has been well documented.^[^ ^36^ ^]^

As a proof‐of‐concept, we further demonstrated that PDS reduced *NOP56* mRNA levels in three cancer cell lines including Hela, A431, and A549 (Figure 6A). The *NOP56*‐G4 sequence is located in the intron region, which is less likely to affect the initial loading of transcription factors to the promoters. In addition, we also examined that r(GGGCCU)_3_GGG did not form RNA G4 in Na^+^ or K^+^ (Figure S29, Supporting Information), suggesting that the inhibited *NOP56* transcription might be attributed to the DNA *NOP56*‐G4 that served a roadblock to impede RNA polymerase processivity (Figure 6B). In addition, our analysis of the previously reported G4 probe‐chromatin immunoprecipitation‐sequencing (G4P‐ChIP‐seq) data acquired in A549 cell line by Zheng et al revealed G4 peaks covering the (GGGCCT)_3_GGG sequence in *NOP56* intron 1 (Figure S30, Supporting Information),^[^ ^37^ ^]^ suggesting the potential of forming *NOP56*‐G4 in cell. In the future work, it will be of interest and significance to investigate the biological roles of the intronic G4 and to develop molecular tools that can specifically target *NOP56*‐G4 such as the CRISPR‐guided G4‐binding protein or ligand.^[^ ^38^ ^]^

In sum, we report the first solution NMR structures of an antiparallel chair‐type G4 from the intron 1 of *NOP56*, namely *NOP56*‐G4 that is composed of two G‐tetrads and one C∙G∙C∙G tetrad capped by a C∙C base pair. We further determined the high‐resolution NMR structures of the *NOP56*‐G4‐PDS binding complex, wherein the ligand stacked at the terminal G‐tetrad through π–π stackings and electrostatic interactions. Moreover, we demonstrated that PDS could reduce *NOP56* mRNA levels in cancer cells, suggesting that the intronic G4 can be a potential druggable target for oncogene regulation and thus expanding the biological significance of G4s. In addition, the high‐resolution structures of free and ligand‐bound *NOP56*‐G4 will benefit DNA structure‐based drug design.

## 4 Experimental Section

### Sample Preparation

Non‐labeled DNA samples were purchased from Sangon Biotech Co. Ltd. (Shanghai, China), and further purified in our laboratory using diethylaminoethyl sephacel anion exchange column and centrifugal desalting devices. The 6% ^13^C/^15^N isotopically labeled DNA sequences were synthesized on a K&A H‐8 synthesizer. The 2′‐deoxyguanosine phosphoramidite (98% ^13^C; 98% ^15^N) was purchased from Cambridge Isotope Laboratories, Inc. USA. The synthesis was initiated by coupling the 3′‐terminal deoxyribonucleotide phosphoramidite to a universal controlled pore glass (Suzhou GenePoly Co., Ltd, China, cat #SP‐C001) filled in a column on the synthesizer. After synthesis, the DNA sequence was cleaved from the controlled pore glass and deprotected by incubating in ammonium hydroxide at 50 °C for 15 h. The sample was then purified by denature PAGE, electro‐elution, diethylaminoethyl sephacel anion exchange column, and centrifugal desalting devices. The purified DNA samples were quantified using a NanoDrop microvolume spectrophotometer. Pyridostatin (PDS, 4‐(2‐aminoethoxy)‐*N^2^*,*N^6^*‐bis(4‐(2‐aminoethoxy)quinolin‐2‐yl) pyridine‐2,6‐dicarboxamide) was purchased from APExBIO (Houston, TX, USA), which was dissolved in DMSO‐*d*_6_ to a stock concentration of 50 mm.

### CD Experiments

CD spectra were recorded on a Chirascan V100 spectropolarimeter. CD samples contained 20 µm DNA in 1 mm sodium phosphate (NaPi) buffer (pH = 5, 6 or 7). The CD spectra were acquired using a 0.5 mm path length quartz cuvette and 1 nm bandwidth at 25 °C. The blank correction was made by subtracting the buffer spectrum. For each sample, an average of three scans was taken. For CD melting experiments, the CD intensity at 295 nm was recorded from 15 to 90 °C at a heating rate of 1 °C min^−1^. Thermodynamic parameters were determined by fitting the CD melting curves using a two‐state transition model.^[^ ^39^ ^]^

### NMR Experiments

NMR samples contained 0.2 mm DNA (for 1D experiments) or 0.5 mm DNA (for 2D experiments) in 1 mm NaPi (pH = 5, 6, or 7). NMR spectra were acquired on a Bruker AVANCE 600 MHz spectrometer and analyzed using TopSpin software. Excitation sculpting and pre‐saturation water suppression methods were used for 10% D_2_O and 99.96% D_2_O samples, respectively. 2D NOESY experiments were conducted with mixing times of 200 ms for 10% D_2_O solvent and 300 ms for 99.96% D_2_O solvent. DQF‐COSY, TOCSY with a mixing time of 75 ms, ^1^H‐^13^C HSQC with a ^1^ *J*_(C,H)_ of 180 Hz, and ^1^H‐^15^N HSQC spectra with a ^1^ *J*_(N,H)_ of 90 Hz were acquired. The 2D NOESY, DQF‐COSY, and TOCSY spectra of free *NOP56*‐G4 in the 99.96% D_2_O solvent were acquired at 30 °C, and all other 1D and 2D NMR spectra were acquired at 25 °C.

### NMR Restraints and Structural Calculations

For structural calculation of free *NOP56*‐G4, the NMR sample containing 0.5 mm DNA in 1 mm NaPi (pH 5) was used. For structural calculation of the *NOP56*‐G4‐PDS complex, the NMR sample containing 0.5 mm DNA, 1.5 mm PDS, 1 mm NaPi (pH 7) was used. NOE‐derived distance restraints were classified as strong (1.8–4.0 Å), strong or medium (2.5–4.5 Å), medium (3.0–5.0 Å), medium or weak (3.5–5.5 Å) and weak (4.0–6.0 Å) according to NOE intensities. A wider range of 1.8–6.0 Å was applied for seriously overlapped NOEs. Glycosidic torsion angles of 90–330° for *anti* base orientation and ‐90‐90° for *syn* base orientation were applied, based on analysis of intranucleotide H6/H8–H1′ NOE intensities and guanine C8 chemical shifts. Chirality restraints for C1′, C3′, and C4′ atoms were generated using Amber. The force constants of hydrogen bond distance, NOE‐derived distance, hydrogen bond angle, backbone, hydrogen bond planarity, G‐tetrad planarity, and chirality restraints were 100, 100 kcal·mol^−1^·Å^−2^, 100, 200, 200, 200, and 100 kcal·mol^−1^·rad^−2^, respectively.

For structural calculation of *NOP56*‐G4‐PDS complex, the partial atomic charges of PDS were obtained by restrained electrostatic potential calculation using Gaussian09 package at the level of HF/6‐31g^*^. Distance restraints of 3.0–6.0 Å were applied for NOEs between *NOP56*‐G4 and PDS. The force constants of PDS‐G4 distance, hydrogen bond distance, NOE‐derived distance, hydrogen bond angle, backbone, hydrogen bond planarity, PDS planarity, G‐tetrad planarity, and chirality restraints were 100, 100, 200 kcal·mol^−1^·Å^−2^, 100, 200, 200, 200, 200, and 25 kcal·mol^−1^·rad^−2^, respectively.

Restrained simulated annealing was performed on AMBER 22^[^ ^40^ ^]^ using bsc1 force field^[^ ^41^ ^]^ in implicit water as previously described.^[^ ^42^ ^]^ The initial structure was based on NOEs and generated using UCSF Chimera.^[^ ^43^ ^]^ For rMD simulations, the system temperature was raised from 300 to 600 K in the first 5 ps, maintained at 600 K for 20 ps, decreased to 300 K within 5 ps, and maintained at 300 K for 5 ps. After the restrained simulated annealing, the set of structural coordinates were subjected for restrained energy minimization by 200 steps of the steepest descent, and conjugated gradient minimization steps until the energy gradient difference between successive minimization steps was smaller than 0.1 kcal∙mol^−1^∙Å^−2^. A total of 500 independent restrained simulated annealing with random starting velocities and energy minimization experiments were performed to obtain 500 initial structures. Twenty structures with the lowest total energies were solvated with TIP3P water and neutralized by Na^+^ cations (the 21‐nt DNA chain was neutralized by 20 Na^+^ ions). The system was first subjected to an energy minimization using 5000 steps of steepest descent, followed by a constant‐volume periodic boundary rMD simulation for 25 ps during which the temperature was increased from 0 to 300 K. Then a 25‐ps constant‐pressure periodic boundary rMD simulation was carried out at 300 K using the Langevin thermostat. The production was performed at 300 K with constant pressure for 1 ns. Finally, a restrained energy minimization was performed using 200 steps of steepest descent, followed by conjugate gradient minimizations until the energy gradient difference between successive minimization steps was smaller than 0.1 kcal·mol^−1^·Å^−2^. Ten structures with the lowest total energies were selected as the final representative ensemble. The RMSD values were calculated using the *suppose* module of AMBER. Structural figures were prepared using PyMOL.

### Unrestrained MD Simulations

Three structures of the free *NOP56*‐G4 with the lowest total energies were subjected to unrestrained MD simulations following established methods^[^ ^6^, ^29^ ^]^ on AMBER 22. After adding 20 Na^+^ to neutralize the phosphate backbone charges, an additional Na^+^ was placed between the G2·G21·G14·G9 and G3·G8·G15·G20 tetrads. The system was then solvated by TIP3P water in a 10‐Å cuboid box. Initial minimizations were carried out with 2000 steps of steepest descent followed by conjugated gradient, during which the DNA positions were fixed. Then the system was heated to 300 K under constant volume in 50 ps, followed by a 50‐ps constant‐pressure relaxation using Langevin thermostat at 300 K. The final production was performed at 300 K without restraints on DNA for 500 ns.

### Native PAGE Experiments

Native PAGE was performed using 20% polyacrylamide gels, supplemented with 1× TBE buffer. DNA loading samples contained 0.2 mm DNA in 1 mm NaPi (pH 7), 0/10/70/150 mm NaCl. The PAGE experiments were conducted at ≈10 °C. DNA bands were visualized by staining the gels with stains‐all solution.

### ITC Experiments

The ITC experiments were performed at room temperature using ITC200 (Malvern Panalytical). The PDS stock solution (200 µM) was titrated from a rotating syringe (750 rpm) into a sample cell containing 300 µL of *NOP56*‐G4 solution (10 µm) with a total of 15 injections (0.4 µL for the first injection and 2.0 µL for the remaining injections). The duration of each injection was 4 s and the delay between injections was 120 s. This initial injection was not used in the fitting. The buffer solutions for ITC experiments contained 1 mm NaPi (pH 7.0). The dissociation constant was determined by three replicative experiments.

### DNA Polymerase Stop Assay

The 1:1 mixture of template and Cy5‐labeled primer (sequences are shown in Table S1, Supporting Information) was annealed in 100 mm KCl by heating at 95 °C for 4 min and cooling to room temperature for 2 h. PDS was added into the annealed sample at target concentrations, and the mixture was incubated overnight. Primer extension was performed for 2 h at 37 °C in 1X reaction buffer (100 mm KCl, 50 mm NaCl, 10 mm Tris‐HCl pH 7.9, 10 mm MgCl_2_ and 1 mm DTT) containing 50 µm primer‐template, 1.5 mm dNTPs (New England Biolabs), and 0.3 U/µL KF with 5′ to 3′ polymerase and 3′ to 5′ exonuclease activities (New England Biolabs). The extended products were resolved using 10% denature PAGE and visualized by Tanon mini space 3000 gel image analysis system.

### RT‐qPCR Experiments

The Hela, A431, and A549 cell lines were purchased from the American Type Culture Collection (ATCC). A431 and A549 cells were grown in DMEM medium supplemented with 10% FBS (Gibco, USA) and 1% penicillin‐streptomycin solution at 37 °C cell incubator. Hela cells were cultured in MEM medium supplemented with 10% FBS and 1% antibiotic. Cells were seeded in a 6‐well plate and treated with different concentrations of PDS for 24 h. Then cells were collected and the total RNA was extracted using TRIzol extraction methods. RNA was reverse transcribed using the PrimeScript™ RT reagent Kit (Takara, Japan) according to the manufacturer's instructions. The TB Green Premix Ex Taq (Takara, Japan) was selected as the amplification reagent and the qPCR was performed using Lightcycler 480 (Roche, Germany). The *β‐actin* mRNA level was used as an endogenous control and 2^−ΔΔCt^ method was used analyze the mRNA levels of *NOP56*. Each experiment was performed in independent triplicate. The primer sequences are summarized in Table S1 (Supporting Information).

### Cell Viability Assay

The cytotoxicity of PDS was assessed using Cell Counting Kit‐8 (CCK‐8). In brief, Hela, A431, and A549 cells were plated into 96‐well plates at a concentration of 5.0×10^3^ cells/well and cultured at the standard condition (37 °C, with 5% CO_2_) mentioned above for 24 h. Then cells were treated with different gradient concentrations of PDS and cultured at 37 °C for 24 h. The cells were then treated with WST‐8 [2‐(2‐methoxy‐4‐nitrophenyl)‐3‐(4‐nitrophenyl)‐5‐(2,4‐disulfophenyl)‐2H‐tetrazolium sodium salt] (Beyotine Biotechnology, China) for 2 h at 37 °C, and the absorbance at 450 nm was determined by a microplate reader. The IC_50_ values were determined from the sigmoidal dose‐response curves using GraphPad Prism 9.0.

### Statistical Analysis

All statistical analyses were performed using GraphPad Prism 9.0 software. Data were presented as mean ± SEM by three replicative experiments for cell experiments, and mean ± S.D. by three replicative experiments for CD melting and ITC experiments. For structural analysis, data were represented as mean ± S.D from ten representative solution NMR structures. One‐way analysis of variance (ANOVA) was performed for multiple comparisons followed by Dunn's multiple comparison tests. For IC_50_ analysis, the data were log transformed to normalized distributions. For all data, statistical significance was set at *p* value less than 0.05 (^*^ *p* <0.05, ^**^ *p* <0.01, ^***^ *p* <0.001).

## Conflict of Interest

The authors declare no conflict of interest.

## Supporting information



Supporting Information

## Data Availability

The coordinates and chemical shifts of the free *NOP56*‐G4 and the *NOP56*‐G4‐PDS complex are available from the Protein Data Bank (PDB IDs: 8XGP; 9JX5) and Biological Magnetic Resonance Bank (BMRB IDs: 36625; 36698), respectively.
