# Supplementary material for: Structural Insights into an Antiparallel Chair‐Type G‐Quadruplex From the Intron of NOP56 Oncogene
Source: Adv Sci (Weinh). 2025 Mar 6;12(16):2406230. doi: 10.1002/advs.202406230 (PMC12021085; doi:10.1002/advs.202406230)
Supplement: Supplementary file 1 — Supporting Information [file ADVS-12-2406230-s001.pdf]

ADVANCED SCIENCE
Open Access

Supporting Information

for *Adv. Sci.*, DOI 10.1002/advs.202406230

Structural Insights into an Antiparallel Chair-Type G-Quadruplex From the Intron of *NOP56* Oncogene

Zhenzhen Yan, Axin He, Liqi Wan, Qian Gao, Yan Jiang, Yang Wang, Ercheng Wang, Changling Li, Yingquan Yang, Yingjie Li, Pei Guo and Da Han**

Supporting Information

Structural Insights into an Antiparallel Chair-Type G-Quadruplex from the Intron of *NOP56* Oncogene

Zhenzhen Yan[1#], Axin He[1,2#], Liqi Wan[1,2#], Qian Gao[1,3], Yan Jiang[1], Yang Wang[1], Ercheng Wang[4], Changling Li[5], Yingquan Yang[1], Yingjie Li[6], Pei Guo[1], Da Han[1,2]**

[1] Zhejiang Cancer Hospital, Hangzhou Institute of Medicine (HIM), Chinese Academy of Sciences, Hangzhou, Zhejiang 310022, China.

[2] Institute of Molecular Medicine (IMM) Renji Hospital, School of Medicine Shanghai Jiao Tong University, Shanghai 200127, China.

[3] Hangzhou Institute for Advanced Study, University of Chinese Academy of Sciences, Hangzhou, Zhejiang 310013, China.

[4] Zhejiang Laboratory, Hangzhou, Zhejiang 311100, China.

[5] College of Chemistry and Materials Science, Shanghai Normal University, Shanghai 200234, China.

[6] Department of Pharmacology, School of Basic Medical Sciences, Peking University Health Science Center, Beijing 100191, China.

[#] Authors who contributed equally.

* Corresponding authors. Email: dahan@sjtu.edu.cn; guopei@ibmc.ac.cn

Table S1. DNA and RNA sequences used in this study.

Name	Sequence
**DNA sequences used for NMR, PAGE, CD and ITC experiments**	
NOP56-G4	5′-GGGCCTGGGCCTGGGCCTGGG-3′
C5T	5′-GGGCTTGGGCCTGGGCCTGGG-3′
C10T	5′-GGGCCTGGGTCTGGGCCTGGG-3′
C11T	5′-GGGCCTGGGCTTGGGCCTGGG-3′
C17T	5′-GGGCCTGGGCCTGGGCTTGGG-3′
C17A	5′-GGGCCTGGGCCTGGGCATGGG-3′
21nt-A	5′-CAACAGTCACACGGCATCTCG-3′
21nt-B	5′-CGAGATGCCGTGTGACTGTTG-3′
**RNA sequence used for NMR experiments**	
r(GGGCCU)$_3$GGG	5′-GGGCCUGGGCCUGGGCCUGGG-3′
**DNA sequences used for DNA polymerase stop assay**	
NOP56-G4 template	5′-GGGCCTGGGCCTGGGCCTGGGTTTTTTTATAGTGAGTCGTATTA -3′
Non-G4 template	5′-GAGCCTGAGCCTGAGCCTGAGTTTTTTTATAGTGAGTCGTATTA -3′
Primer	5′-(Cy5)TAATACGACTCACTATA-3′
Full DNA reference	5′-(Cy5)TAATACGACTCACTATAAAAAAACCCAGGCCCAGGCCCAGGCCC-3′
Stalled DNA reference	5′-(Cy5)TAATACGACTCACTATAAAAAAA-3′
**DNA sequences used for RT-qPCR experiment**	
NOP56 forward primer	5′-GTGAAGGGTCTGACCGATCTG-3′
NOP56 reverse primer	5′-TAACTTTGGCACGGGAATAGC-3′
β-actin forward primer	5′-CTTCCAGCCTTCCTTCCTGG-3′
β-actin reverse primer	5′-TTCTGCATCCTGTCGGCAA-3′

NMR: nuclear magnetic resonance; PAGE: polyacrylamide gel electrophoresis; CD: circular dichroism; ITC: isothermal titration calorimetry; RT-qPCR: real-time quantitative polymerase chain reaction.

Table S2. ^{1}H chemical shifts (ppm) of the free *NOP56*-G4[a].

Residue	H1	H41/H42	H5/H7	H6/H8	H1′	H2′	H2″	H3′	H4′
G1	[b]	-	-	7.62	5.88	2.51	2.78	4.86	[c]
G2	11.48	-	-	7.47	5.98	3.33	2.84	4.96	[c]
G3	12.13	-	-	8.13	5.73	2.73	2.59	5.04	[c]
C4	-	9.31/8.49	5.08	7.33	6.09	2.57	2.57	4.36	[c]
C5	-	8.61/7.58	5.50	7.21	5.38	1.30	2.36	4.47	[c]
T6	-	-	1.53	7.40	6.06	2.12	2.29	4.73	[c]
G7	13.01	-	-	7.92	6.24	2.60	2.90	4.82	4.48
G8	11.42	-	-	7.34	5.94	3.21	2.74	5.02	[c]
G9	12.02	-	-	7.76	6.07	[b]	[b]	5.03	[c]
C10	-	[b]	6.13	7.99	6.27	2.71	2.40	4.95	[c]
C11	-	[b]	6.15	7.76	6.06	2.14	2.37	4.50	[c]
T12	-	-	1.43	7.11	5.85	1.76	1.67	4.49	[c]
G13	[b]	-	-	7.65	5.81	2.81	2.81	5.06	[b]
G14	11.67	-	-	7.38	6.01	3.45	2.82	4.92	[c]
G15	12.21	-	-	8.17	5.79	2.78	2.65	5.06	[c]
C16	-	9.50/8.53	5.10	7.34	6.12	2.55	2.55	4.44	[c]
C17	-	8.61/7.58	5.52	7.20	5.43	1.30	2.36	4.47	[c]
T18	-	-	1.50	7.34	6.02	2.11	2.27	4.72	[c]
G19	13.07	-	-	7.91	6.20	2.60	2.89	4.82	4.48
G20	11.28	-	-	7.33	5.91	3.21	2.74	5.02	[c]
G21	11.91	-	-	7.93	6.14	2.67	2.51	4.77	4.26

[a)] Chemical shifts of labile and non-labile protons were measured at 25 and 30 °C (pH 5), respectively.
[b)] Signals were too broadened or too weak to be observed.
[c)] Resonances could not be assigned due to weak $^{3}J_{H3'\text{-}H4'}$ couplings or signal overlaps.

Table S3. A summary of the inter-nucleotide NOE-derived distance restraints for the flanking residue (G1) and loop residues (C5, T6, C10, C11, T12, G13, C17, T18) of the free *NOP56*-G4.

Residue	Atom	Residue	Atom	Distance lower bound (Å)	Distance upper bound (Å)
G1	H8	G2	H1	1.8	6.0
G1	H2'	G2	H1	1.8	6.0
G1	H2"	G2	H1	1.8	6.0
G1	H1'	G2	H1	1.8	6.0
G1	H8	G9	H1	1.8	6.0
C5	H6	C4	H2'	1.8	6.0
C5	H6	C4	H2"	1.8	6.0
C5	H6	C4	H3'	1.8	6.0
C5	H6	C4	H1'	3.5	5.5
C5	H5	C4	H2"	1.8	6.0
C5	H5	C4	H41	1.8	6.0
C5	H3'	T6	H7	1.8	6.0
C5	H2"	T6	H7	1.8	6.0
C5	H2'	T6	H6	3.5	5.5
C5	H2"	T6	H6	2.5	4.5
C5	H3'	T6	H6	3.0	5.0
C5	H1'	T6	H6	1.8	6.0
C5	H2'	G7	H1	4.0	6.0
C5	H1'	G7	H1	1.8	6.0
C5	H6	G7	H1	1.8	6.0
C10	H6	C11	H5	4.0	6.0
C10	H1'	T12	H7	1.8	6.0
C11	H2'	T12	H6	4.0	6.0
C11	H2"	T12	H6	3.0	5.0
C17	H6	C16	H2'	1.8	6.0
C17	H6	C16	H2"	1.8	6.0
C17	H6	C16	H1'	4.0	6.0
C17	H3'	T18	H7	1.8	6.0
C17	H2"	T18	H7	1.8	6.0
C17	H2'	T18	H6	1.8	6.0
C17	H2"	T18	H6	2.5	4.5
C17	H6	G19	H1	1.8	6.0
C17	H1'	G19	H1	1.8	6.0

Table S4. Glycosidic torsion angle (χ) restraints for structural calculation of free *NOP56*-G4.

**Residue**	**χ (°)**
G1	-
G2	-90-90
G3	90-330
C4	90-330
C5	90-330
T6	90-330
G7	90-330
G8	-90-90
G9	90-330
C10	90-330
C11	90-330
T12	90-330
G13	-
G14	-90-90
G15	90-330
C16	90-330
C17	90-330
T18	90-330
G19	90-330
G20	-90-90
G21	90-330

Table S5. Hydrogen bond restraints for structural calculation of free *NOP56*-G4.

For G7-C4 and G19-C16 Watson-Crick base pairs	
**Atoms (G-C)**	**Restraints**
H1-N3	1.84-2.04 Å
H21-O2	1.75-1.95 Å
N1-N3	2.85-3.05 Å
O6-H41	1.80-2.00 Å
O6-N4	2.81-3.01 Å
O6···H41-N4	170-190°
N1-H1···N3	170-190°
N2-H21···O2	170-190°

Table S6. G-tetrad planarity restraints for structural calculation of free *NOP56*-G4[a].

Atoms	Angle (°)
N9, N7, N1, N3	355-365
C5, C2, N7, C4	355-365
C2, C8, C2, C8	355-365

[a)] Three planarity restraints were applied for every two adjacent guanines in each G-tetrad.

Table S7. Chirality restraints for structural calculation of free *NOP56*-G4[a].

Atoms	Angle (°)
C2′, O4′, N1/N9, H1′	60-80
O3′, C2′, C4′, H3′	60-80
C3′, C5′, O4′, H4′	60-80

[a)] The chirality restraints were generated using AMBER. The three chirality restraints were applied for each of the 21 nucleotides.

Table S8. ^{1}H chemical shifts (ppm) of the *NOP56*-G4-PDS complex[a].

Residue	H1	H41/H42	H5/H7	H6/H8	H1′	H2′	H2″	H3′	H4′
G1	b	-	-	7.90	6.20	2.80	2.80	5.05	c
G2	11.12	-	-	7.51	6.08	3.63	2.87	b	c
G3	11.99	-	-	7.97	5.64	2.60	2.53	4.95	4.44
C4	-	9.27/8.63	5.01	7.24	6.05	2.51	2.51	b	c
C5	-	8.48/7.30	5.42	7.14	5.37	1.27	2.34	4.44	c
T6	-	-	1.50	7.36	6.02	2.09	2.26	4.70	c
G7	12.96	-	-	7.81	6.04	2.53	2.90	b	c
G8	11.25	-	-	7.11	5.83	3.21	2.76	5.03	4.41
G9	10.80	-	-	8.07	5.74	2.75	2.52	b	c
C10	-	b	5.85	7.81	6.12	2.40	2.17	4.68	c
C11	-	b	5.69	7.64	6.05	2.61	2.31	4.91	4.52
T12	-	-	1.36	7.19	5.81	1.48	2.03	b	c
G13	b	-	-	7.54	6.04	b	b	b	c
G14	11.60	-	-	6.87	5.97	3.46	2.83	4.97	4.59
G15	11.90	-	-	8.04	5.79	2.69	2.65	5.04	4.49
C16	-	9.56/8.74	5.00	7.24	6.13	2.46	2.54	4.48	c
C17	-	8.68/7.49	5.46	7.17	5.37	1.26	2.35	4.44	c
T18	-	-	1.46	7.31	5.99	2.06	2.24	4.67	c
G19	13.16	-	-	7.76	6.14	2.51	2.93	b	c
G20	11.10	-	-	7.32	5.89	3.02	2.76	5.01	4.37
G21	11.22	-	-	7.93	6.04	2.72	2.90	5.11	4.52

a) Chemical shifts were measured at 25 °C (pH 7).
b) Signals were too broadened or too weak to be observed.
c) Resonances could not be assigned due to weak $^{3}J_{H3'\text{-}H4'}$ couplings or signal overlaps.

Table S9. A summary of the inter-nucleotide NOE-derived distance restraints for the flanking residue (G1) and loop residues (C5, T6, C10, C11, T12, G13, C17, T18) of the *NOP56*-G4-PDS complex.

Residue	Atom	Residue	Atom	Distance lower bound (Å)	Distance upper bound (Å)
C5	H6	C4	H2'	1.8	6.0
C5	H6	C4	H2"	1.8	6.0
C5	H6	C4	H1'	4.0	6.0
C5	H3'	T6	H7	1.8	4.0
C5	H1'	T6	H7	1.8	6.0
C5	H2"	T6	H6	1.8	4.0
C5	H2'	T6	H6	2.5	4.5
C5	H1'	T6	H6	3.5	5.5
C11	H1'	T12	H7	4.0	6.0
C11	H6	T12	H1'	4.0	6.0
C17	H6	C16	H2"	1.8	4.0
C17	H6	C16	H2'	2.5	4.5
C17	H6	C16	H1'	4.0	6.0
C17	H3'	T18	H7	1.8	4.0
C17	H1'	T18	H7	1.8	6.0
C17	H2"	T18	H6	1.8	4.0
C17	H2'	T18	H6	3.0	5.0
C17	H1'	T18	H6	3.5	5.5

Table S10. Glycosidic torsion angle (χ) restraints for structural calculation of *NOP56*-G4-PDS complex.

Residue	χ (°)
G1	-
G2	-90-90
G3	90-330
C4	90-330
C5	90-330
T6	90-330
G7	90-330
G8	-90-90
G9	90-330
C10	-
C11	90-330
T12	90-330
G13	-90-90
G14	-90-90
G15	90-330
C16	90-330
C17	90-330
T18	90-330
G19	90-330
G20	-90-90
G21	90-330

Table S11. Hydrogen bond restraints for structural calculation of *NOP56*-G4-PDS.

For G7-C4 and G19-C16 Watson-Crick base pairs	
**Atoms (G-C)**	**Restraints**
H1-N3	1.84-2.04 Å
H21-O2	1.75-1.95 Å
N1-N3	2.85-3.05 Å
O6-H41	1.80-2.00 Å
O6-N4	2.81-3.01 Å
O6···H41-N4	170-190°
N1-H1···N3	170-190°
N2-H21···O2	170-190°

Table S12. G-tetrad planarity restraints for structural calculation of *NOP56*-G4-PDS[a].

**Atoms**	**Angle (°)**
N9, N7, N1, N3	355-365
C5, C2, N7, C4	355-365
C2, C8, C2, C8	355-365

[a)] Three planarity restraints were applied for every two adjacent guanines in each G-tetrad.

Table S13. Chirality restraints for structural calculation of *NOP56*-G4-PDS[a].

Atoms	Angle (°)
C2′, O4′, N1/N9, H1′	60-80
O3′, C2′, C4′, H3′	60-80
C3′, C5′, O4′, H4′	60-80

[a)] The chirality restraints were generated using AMBER. The three chirality restraints were applied for each of the 21 nucleotides.

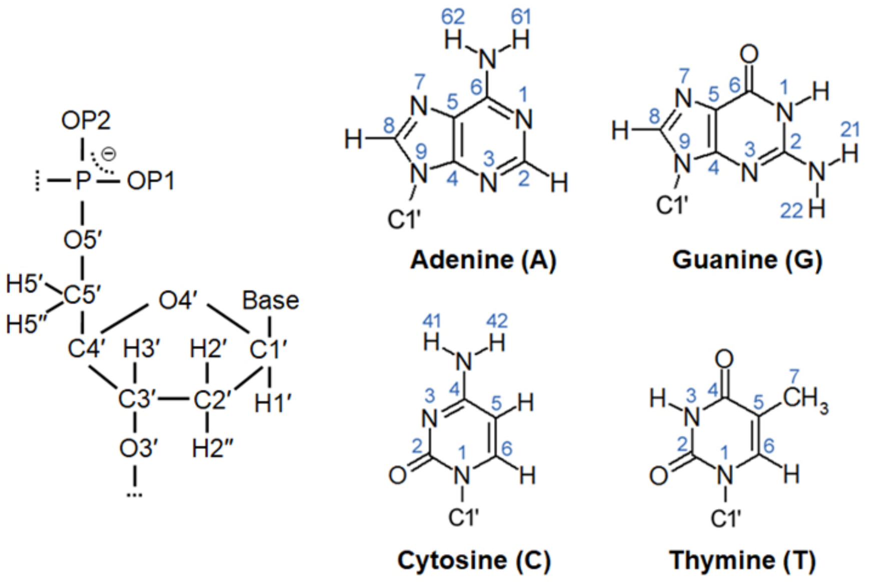

Figure S1. Atom numbering of the four deoxyribonucleotides following the International Union of Pure and Applied Chemistry (IUPAC) guidelines.[1]

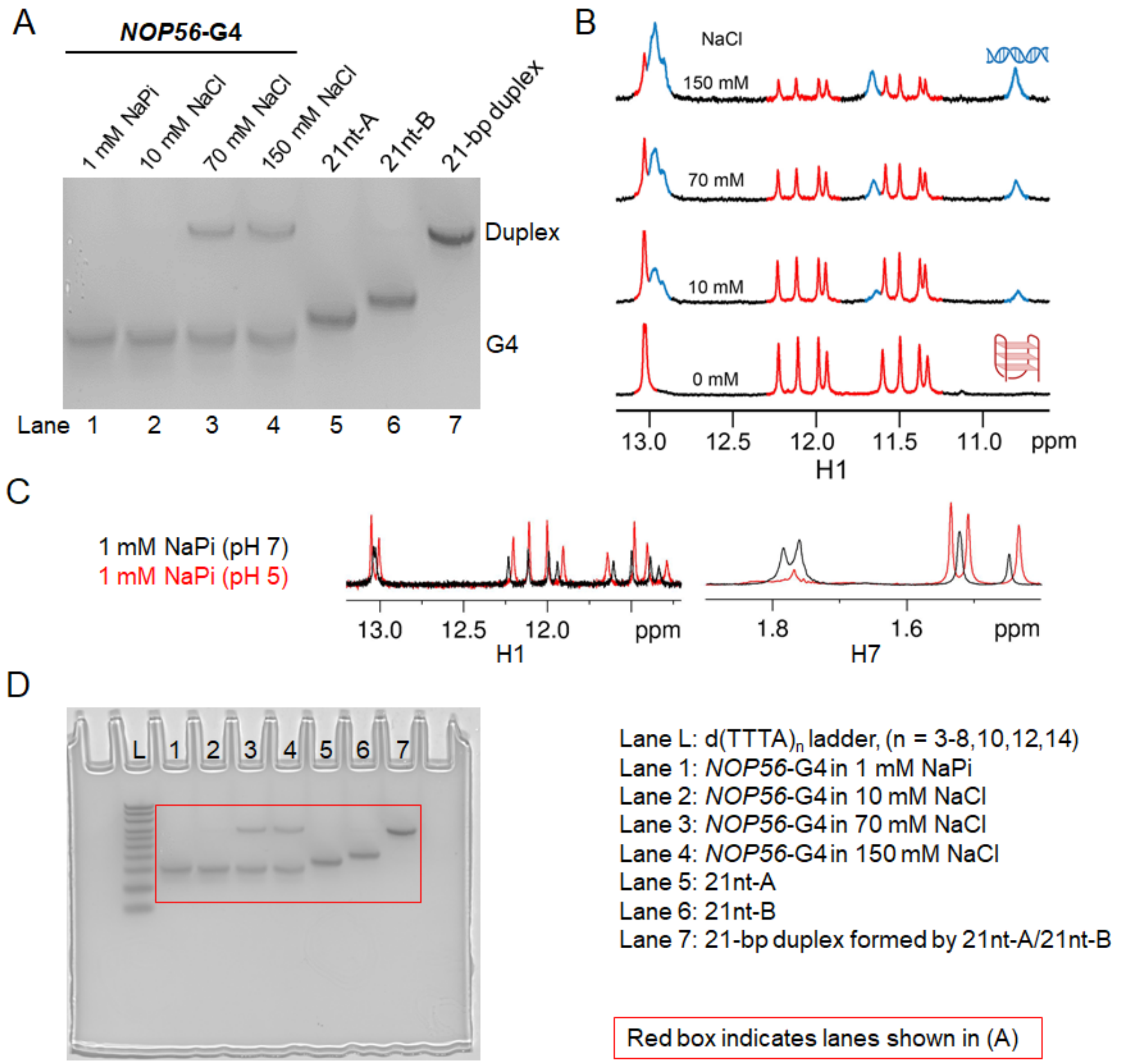

Figure S2. (A) Native PAGE of *NOP56*-G4 with various concentrations of NaCl at pH 7. Lanes 1-4: *NOP56*-G4 with varied NaCl concentrations. Lane 5: 21-nt single stranded 21nt-A (5′-CAACAGTCACACGGCATCTCG-3′). Lane 6: 21-nt single stranded 21nt-B (5′-CGAGATGCCGTGTGACTGTTG-3′). Lane 7: a 21-bp duplex reference formed by 21nt-A/21nt-B in the presence of 20 mM Mg^{2+}, as designed using the NUPACK server.[2] (B) 1D ^{1}H NMR spectra of *NOP56*-G4 with various concentrations of NaCl at pH 7. NMR peaks from G4 and duplex structures are labeled by red and blue colors, respectively. [DNA] = 0.2 mM, [NaPi, pH 7] = 1 mM. (C) Overlaid ^{1}H NMR spectra of *NOP56*-G4 at pH 7 (black) and pH 5 (red). The intensity of H1 signals are increased by eight fold as compared to the H7 region. [DNA] = 0.2 mM, [NaPi] = 1 mM. (D) The original uncropped gel of (A).

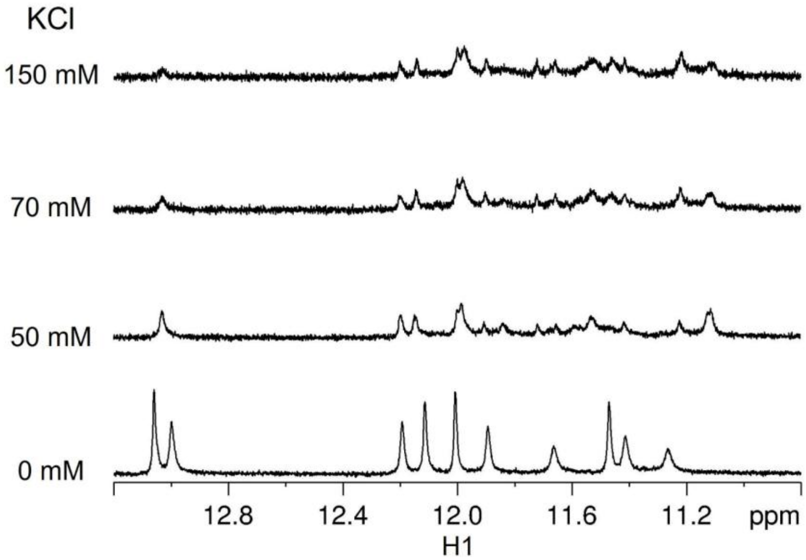

Figure S3. 1D ^{1}H NMR spectra (G H1 region) of *NOP56*-G4 in various concentrations of KCl. NMR signals from *NOP56*-G4 were reduced but still observable in 50/70/150 mM KCl. The additional G H1 signals suggested that apart from the *NOP56*-G4, other conformer(s) also formed in 50/70/150 mM K^+. [DNA] = 0.2 mM, [NaPi, pH 5] = 1 mM, 25 °C.

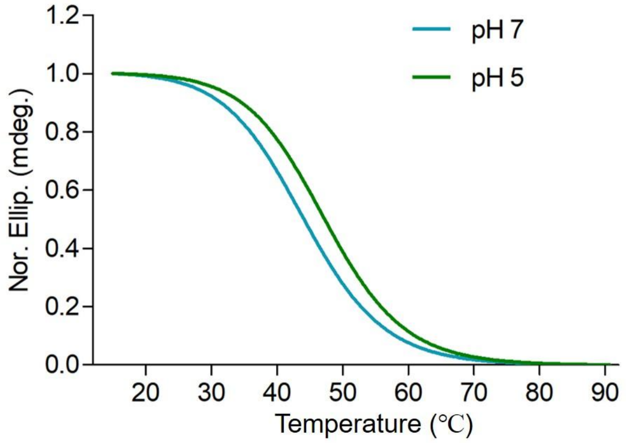

Figure S4. Normalized CD melting curves of *NOP56*-G4 at pH 5 (green) and pH 7 (blue) constructed by measuring CD intensity at 295 nm as a function of temperature. The melting temperature (T_m) of *NOP56*-G4 at pH 5 and pH 7 were determined to be 46.5 ± 0.4 and 44.8 ± 0.7 ℃, respectively. [NaPi] = 1 mM. Data are represented as mean ± S.D. by three replicative experiments.

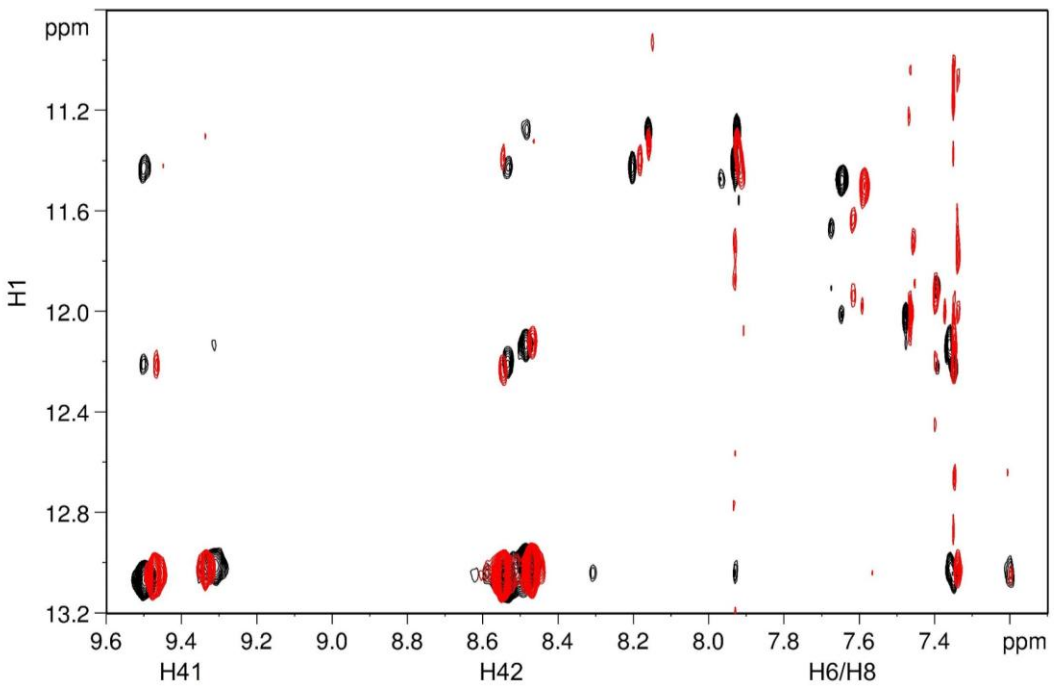

Figure S5. Overlaid nuclear Overhauser effect spectroscopy (NOESY) spectra showing the H1-H41/H42/H6/H8 regions of free *NOP56*-G4 at pH 7 (red) and pH 5 (black). [NaPi] = 1 mM, 25 °C.

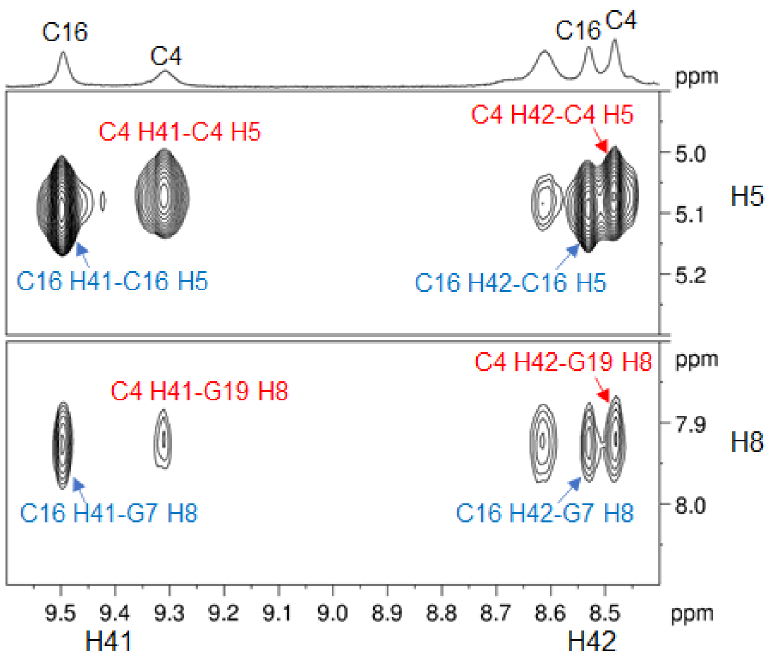

Figure S6. Assignment of C4 and C16 H41/H42 by intra-nucleotide cytosine H5-H41/H42 NOEs (top), and the NOEs of C4 H41/H42-G19 H8 and C16 H41/H42-G7 H8 supporting the C4·G7·C16·G19 tetrad in free *NOP56*-G4 (bottom). NOEs involving C4 and C16 are labeled in red and blue, respectively. [NaPi, pH 5] = 1 mM, 10% D_2O, 25 °C.

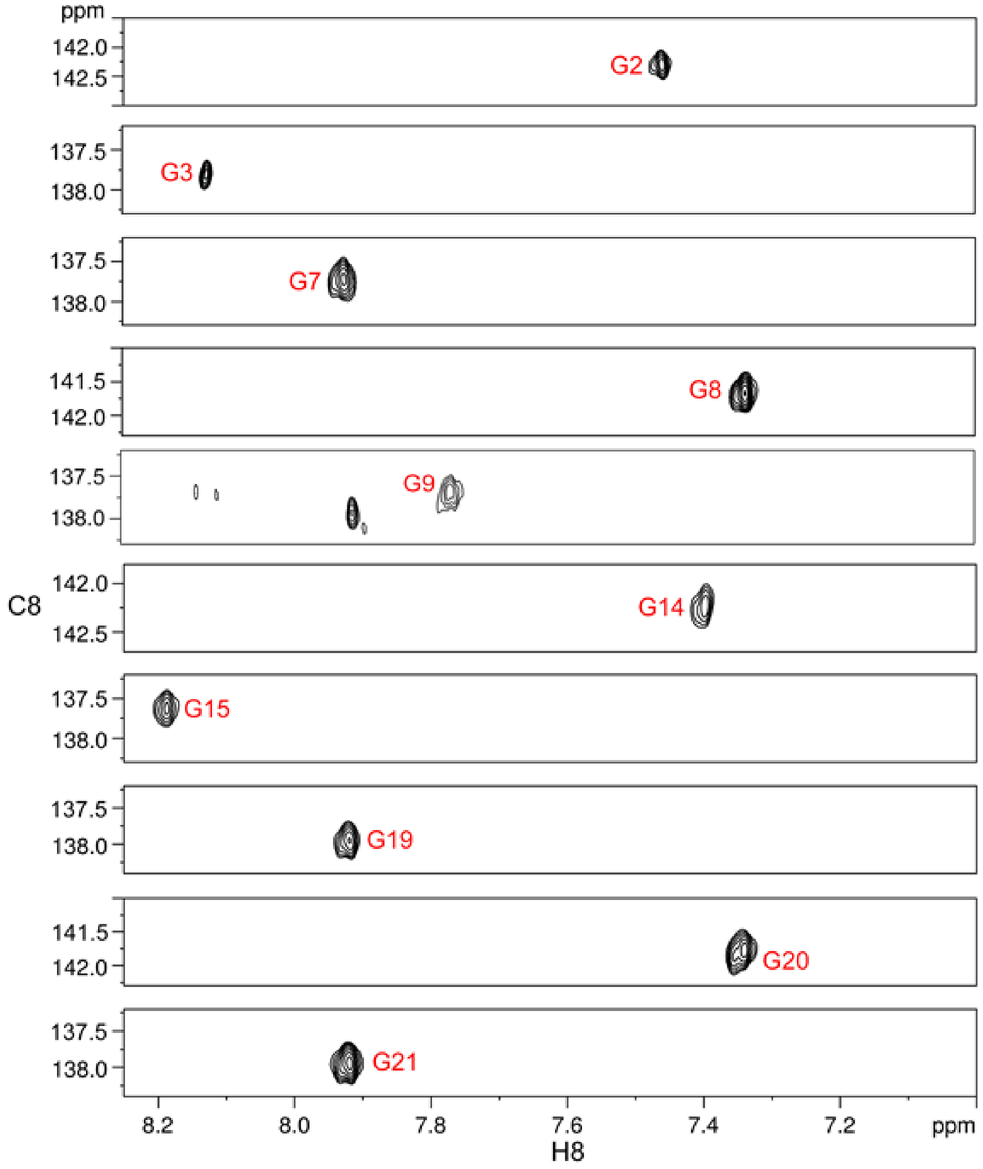

Figure S7. ^{1}H-^{13}C heteronuclear single quantum coherence (HSQC) NMR spectra of 6% ^{13}C/^{15}N guanine-labeled free *NOP56*-G4. [NaPi, pH 5] = 1 mM, 25 °C.

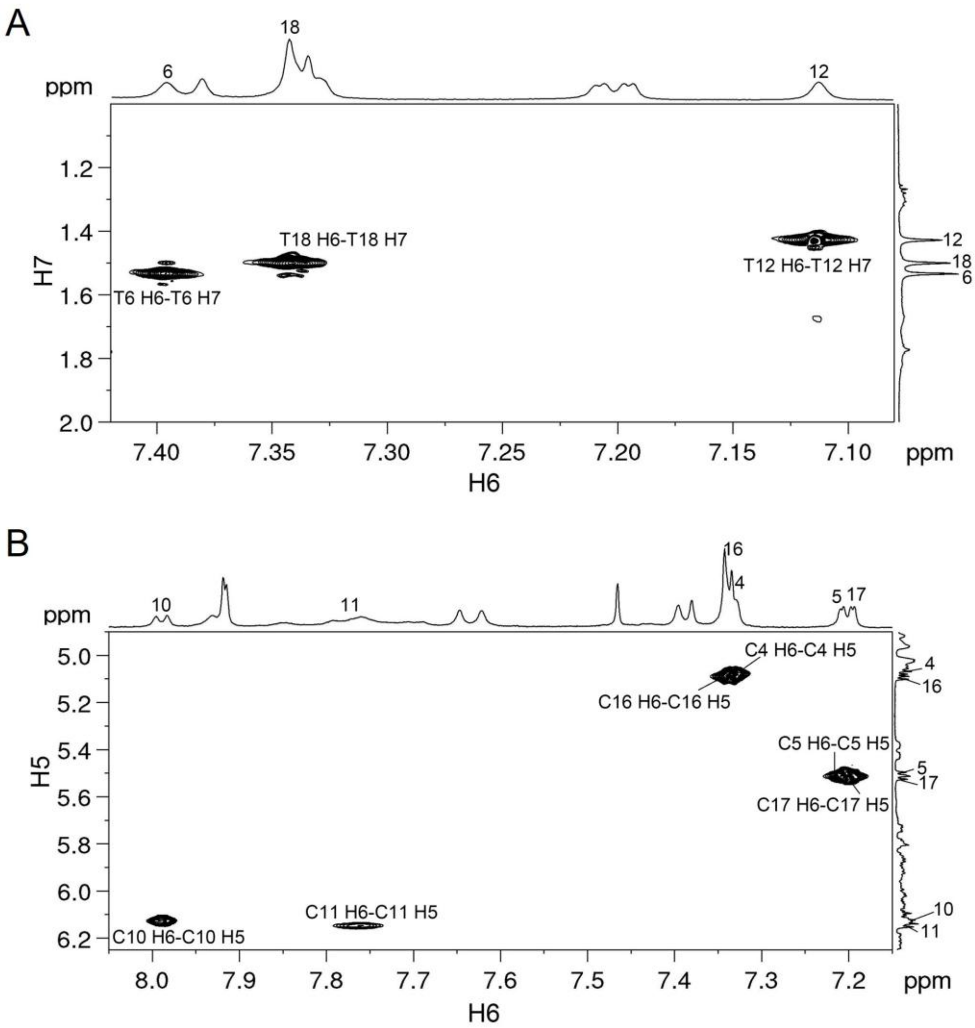

Figure S8. Total correlation spectroscopy (TOCSY) of free *NOP56*-G4 shows the thymine H6-H7 and cytosine H5-H6 correlation peaks. [NaPi, pH 5] = 1 mM, 99.96% D_2O, 30 °C.

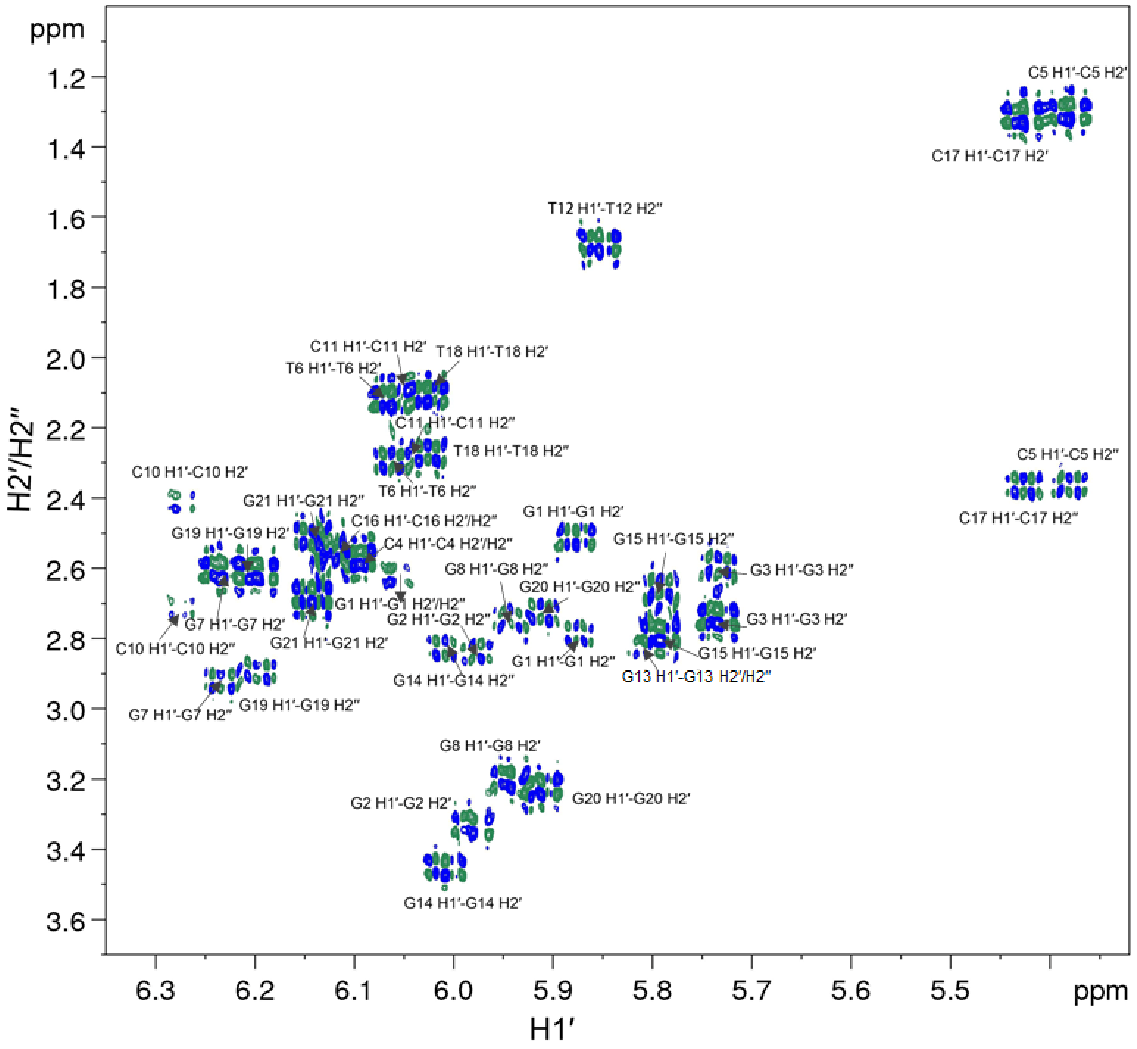

Figure S9. Double-quantum filtered correlation spectroscopy (DQF-COSY) of free *NOP56*-G4 shows the intra-nucleotide H1′-H2′/H2″ correlation peaks. [NaPi, pH 5] = 1 mM, 99.96% D_2O, 30 °C. Phase-sensitive correlation peaks are shown in blue and green colors.

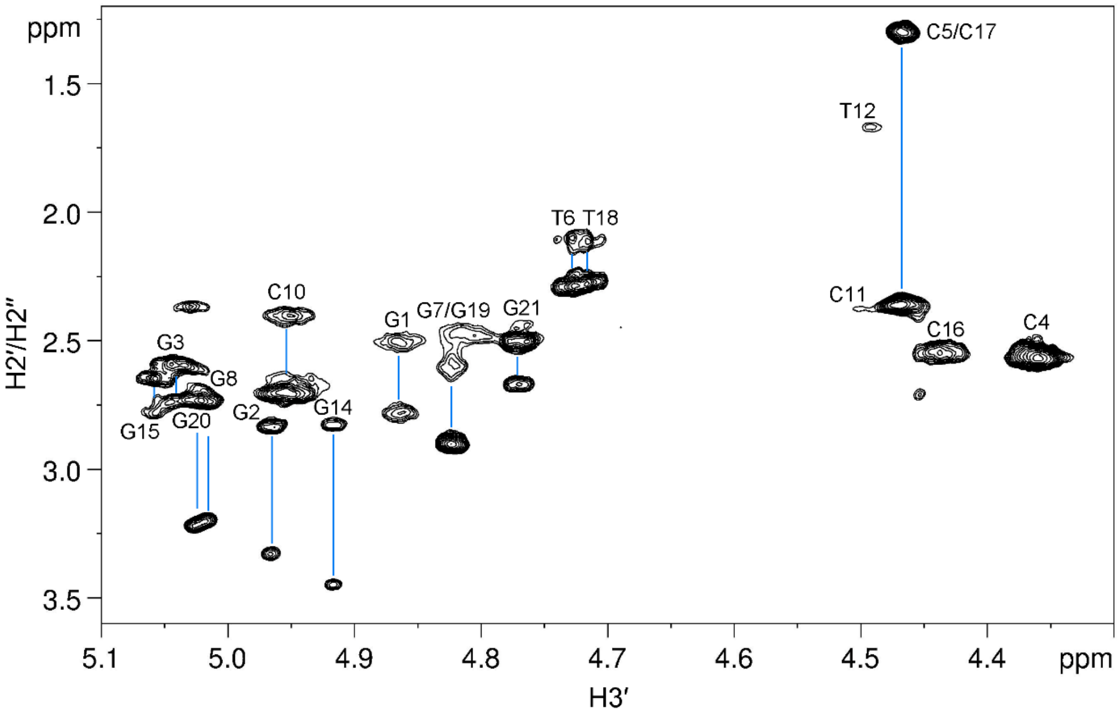

Figure S10. TOCSY spectrum of free *NOP56*-G4 shows H3′ resonance assignment. [NaPi, pH 5] = 1 mM, 99.96% D_2O, 30 °C.

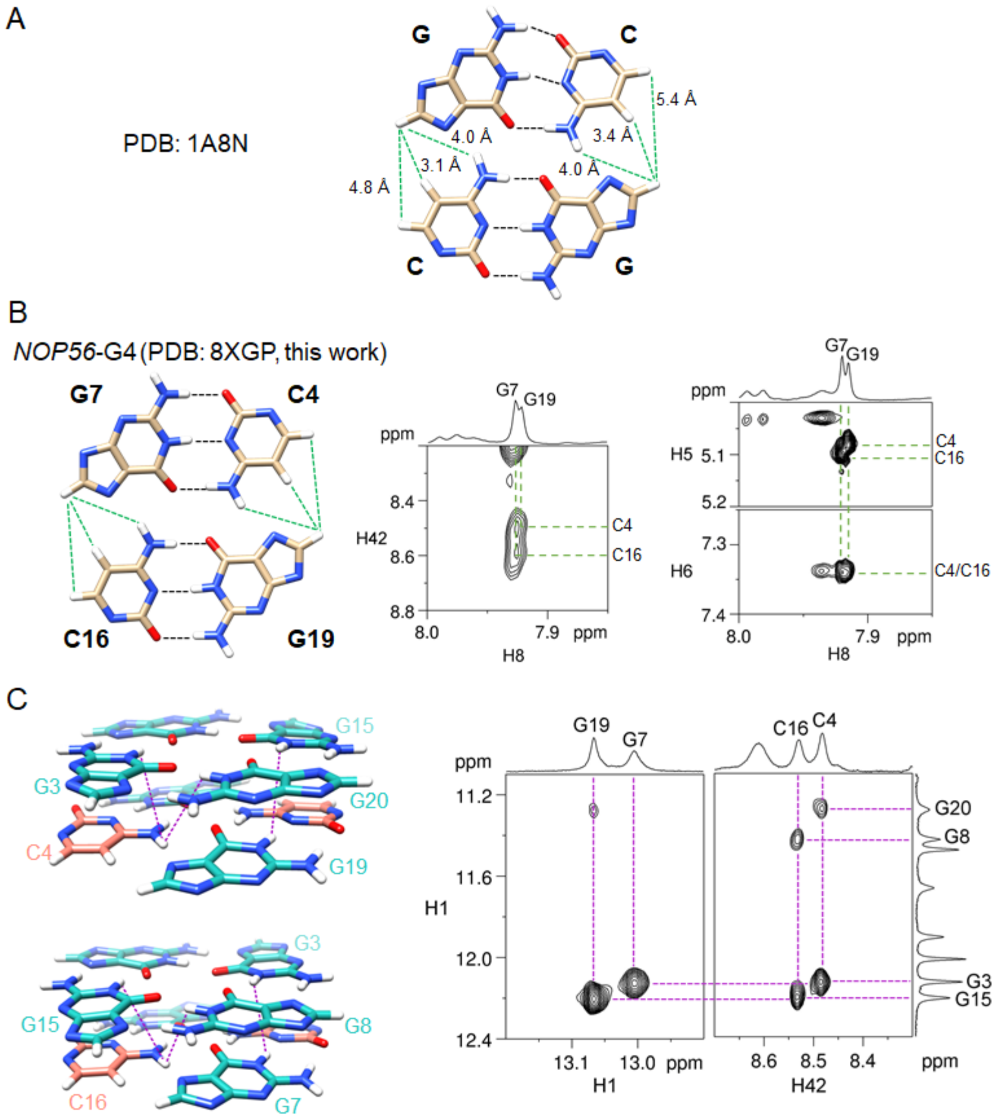

Figure S11. Formation of C4·G7·C16·G19 tetrad in the free *NOP56*-G4. (A) Structure of a reported C·G·C·G tetrad with a direct alignment (PDB ID: 1A8N).[3] The black dotted lines represent hydrogen bonds in Watson-Crick base pairs, and green dotted lines delineate that the distances between guanine H8 from one Watson-Crick base pair and cytosine H42/H5/H6 from the other Watson-Crick base pair are close (within 6 Å). (B) In *NOP56*-G4, the NOEs of G7 H8-C16 H42/H5/H6 and G19 H8-C4 H42/H5/H6 support the spatial proximity of guanine H8 from one Watson-Crick base pair and cytosine H42/H5/H6 from the other Watson-Crick base pair. (C) In *NOP56*-G4, the NOEs of C4 H42-G3/G20 H1, G7 H1-G3 H1, C16 H42-G8/G15 H1 and G19 H1-G15 H1 revealed that all residues in the C4·G7·C16·G19 tetrad stacked with the neighboring G3·G8·G15·G20 tetrad, further consolidating the co-planarity of C4, G7, C16 and G19 and thus the C4·G7·C16·G19 tetrad. The observed NOEs in (B) and (C) indicate proton-proton distance within ~6 Å.

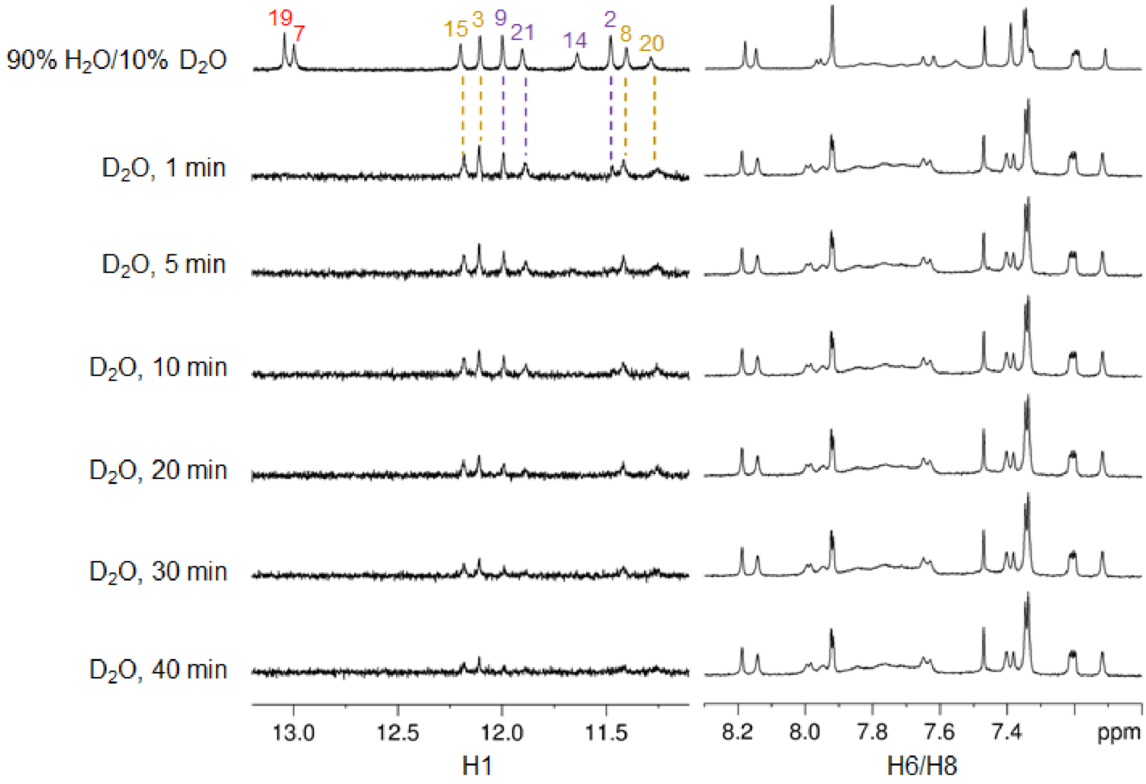

Figure S12. The ^{1}H NMR spectra of *NOP56*-G4 acquired at 1, 5, 10, 20, 30 and 40 min immediately after the DNA sample was dissolved in 99.96% D_2O. H1 signals from the C4·G7·C16·G19, G2·G21·G14·G9 and G3·G8·G15·G20 tetrads are labeled in red, purple and yellow colors, respectively. The signal intensity of the H1 region was amplified by four folds relative to the H6/H8 region. [NaPi, pH 5] = 1 mM, 25 °C.

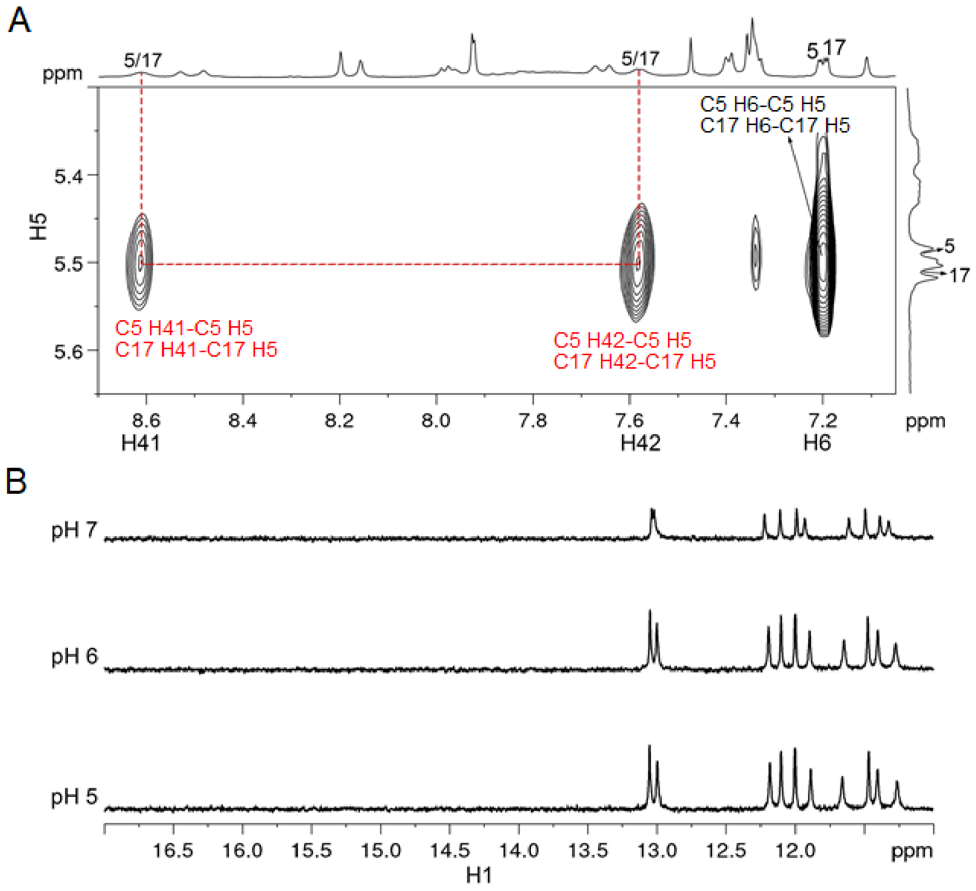

Figure S13. (A) NOESY spectrum of free *NOP56*-G4 shows intranucleotide NOEs of C5 H6-H5, C17 H6-H5 (black), C5 H41-H5, C5 H42-H5, C17 H41-H5 and C17 H42-H5 (red). [NaPi, pH 5] = 1 mM, 10% D_2O, 25 °C. (B) 1D ^1H NMR spectra (imino proton region) of free *NOP56*-G4 at pH 5, 6 and 7. No protonated cytosine H3 signal was observed at 15.0 to 17.0 ppm. [NaPi] = 1 mM, 10% D_2O, 25 °C.

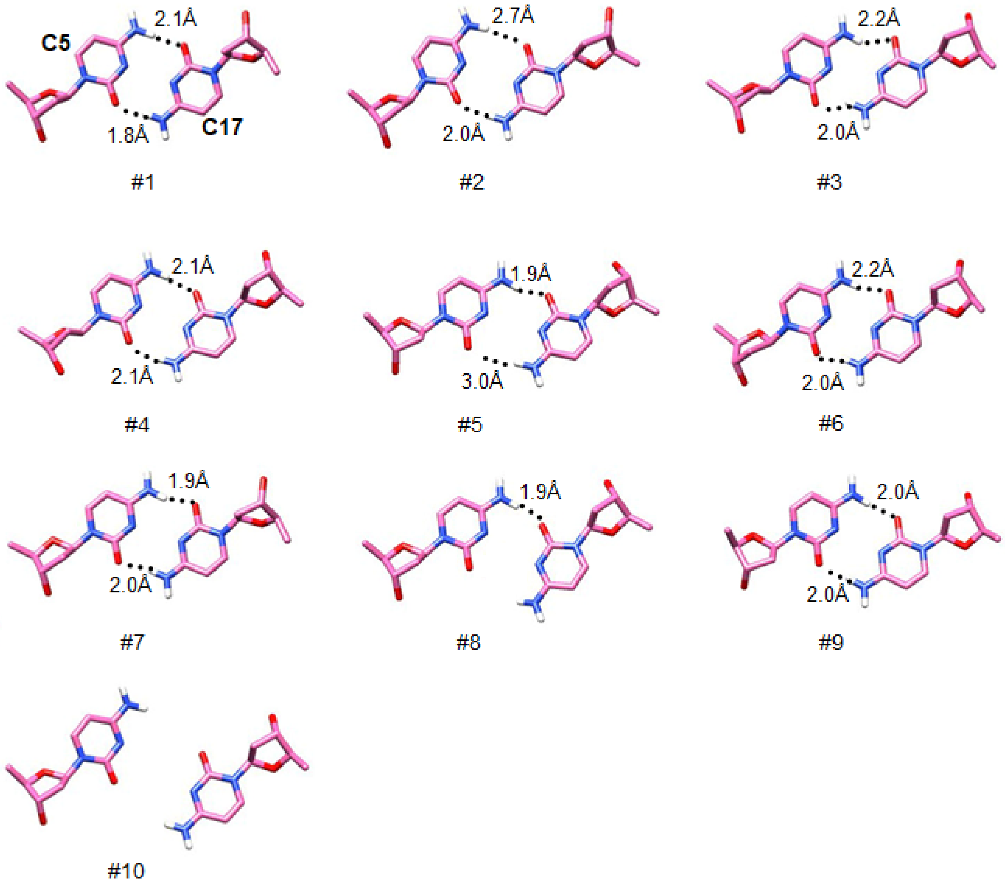

Figure S14. In the ten solution NMR structures of free *NOP56*-G4, C5 and C17 predominantly formed a two-hydrogen-bond base pair as observed in eight structures, one-hydrogen-bond base pair in one structure (#8), and no hydrogen bond in one structure (#10). The dotted line represents the formation of a hydrogen bond (distance between the hydrogen bond donor H41/H42 and acceptor O2 ≤ 3.2 Å).

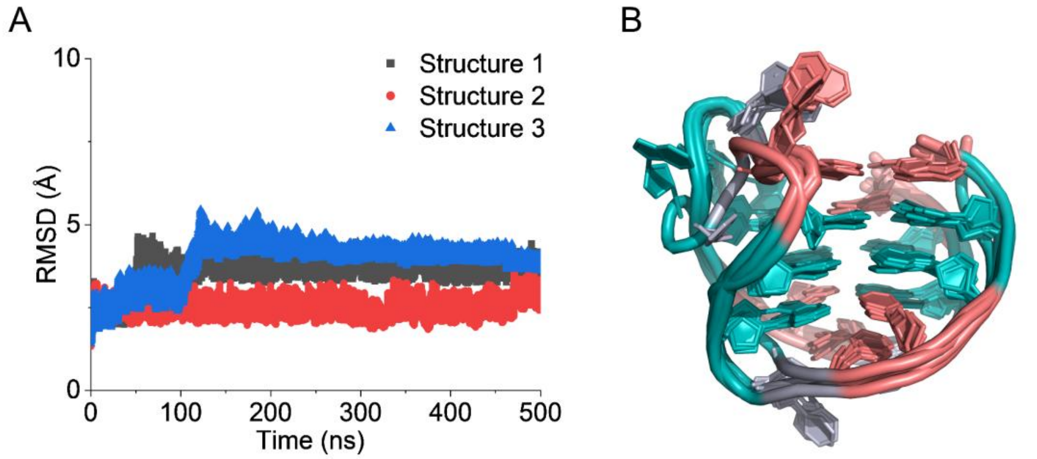

Figure S15. Unrestrained 500-ns MD simulations on *NOP56*-G4. (A) RMSD values of the three independent 500-ns MD simulations. (B) Superposition of structures sampled at 0, 100, 200, 300, 400 and 500 ns from one representative simulation. Guanine, cytosine and thymine residues are colored in green, red and gray, respectively.

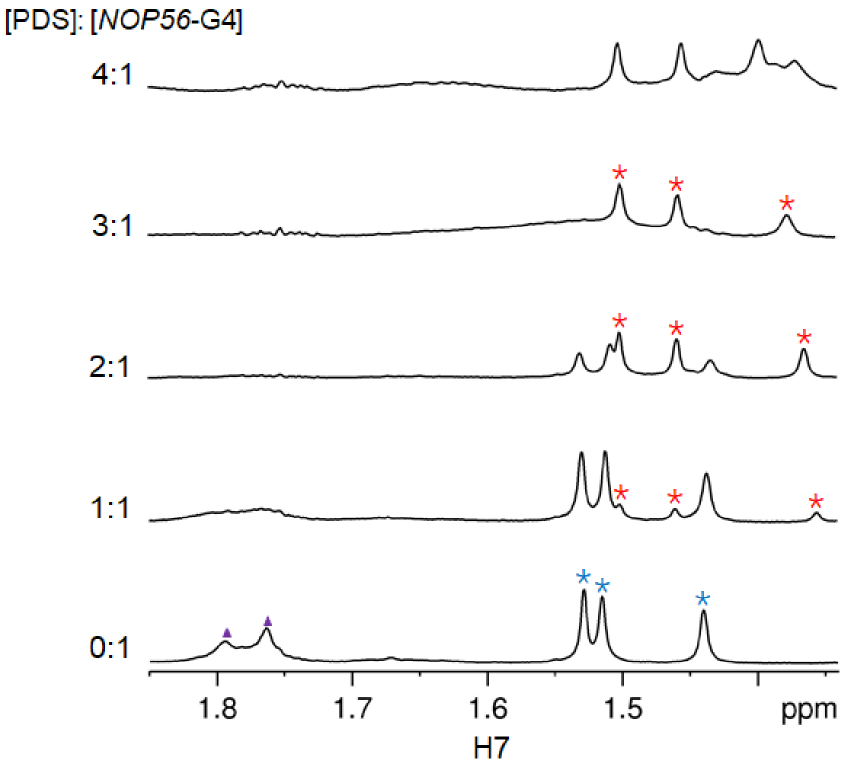

Figure 16. 1D ^{1}H NMR spectra (thymine methyl H7 region) of *NOP56*-G4 upon PDS titration at pH 7. Upon adding PDS, the H7 signals at ~1.75 to 1.80 ppm arising from random coils (purple triangles) and H7 signals at ~1.40 to 1.55 ppm from the free G4 (blue asterisks) vanished, and meanwhile the H7 signals at ~1.35 to 1.50 ppm from *NOP56*-G4-PDS complex increased (red asterisks). [NaPi, pH 7] = 1 mM, 25 °C.

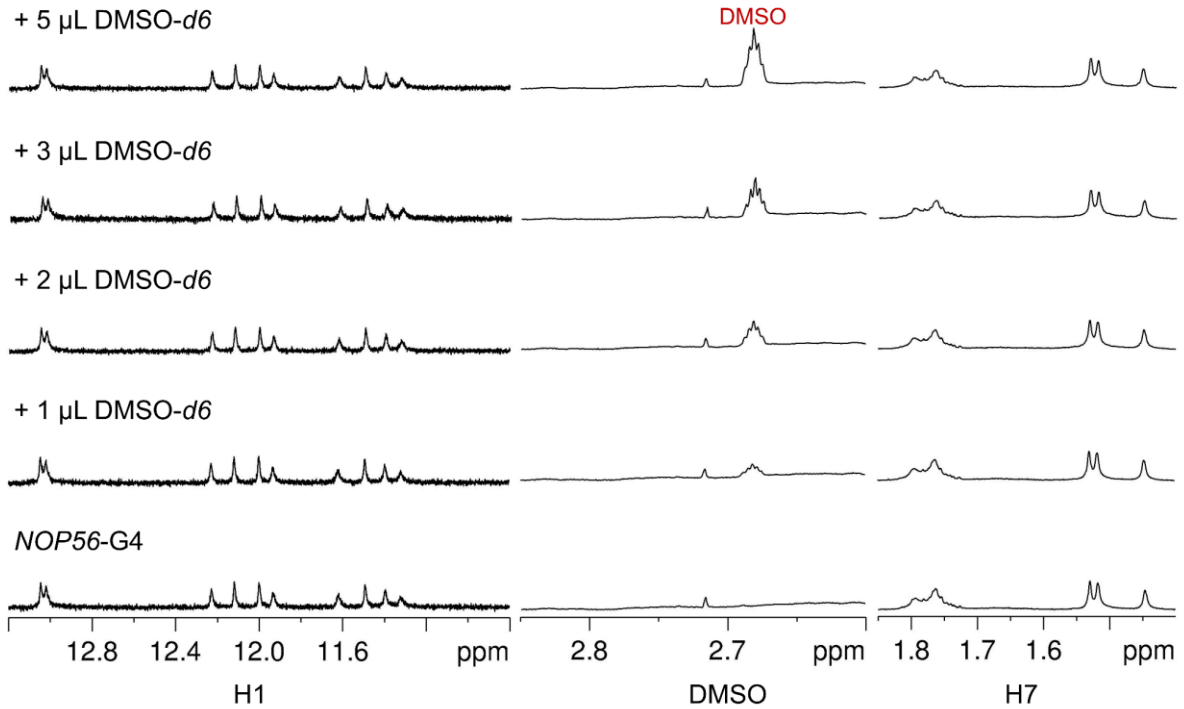

Figure S17. 1D ^{1}H NMR spectra of *NOP56*-G4 upon adding DMSO-*d*6. The H1 region was amplified by eight folds relative to the DMSO and H7 regions. [NaPi, pH 7] = 1 mM, 25 °C.

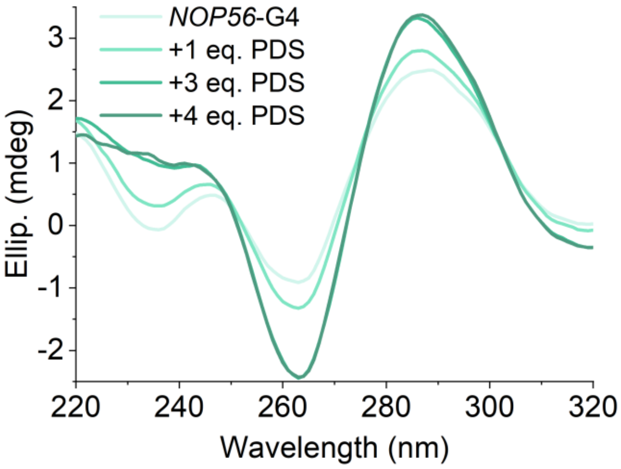

Figure S18. CD spectra of *NOP56*-G4 at 0, 1, 3 and 4 equivalents of PDS. [NaPi, pH 7] = 1 mM, 25 ℃.

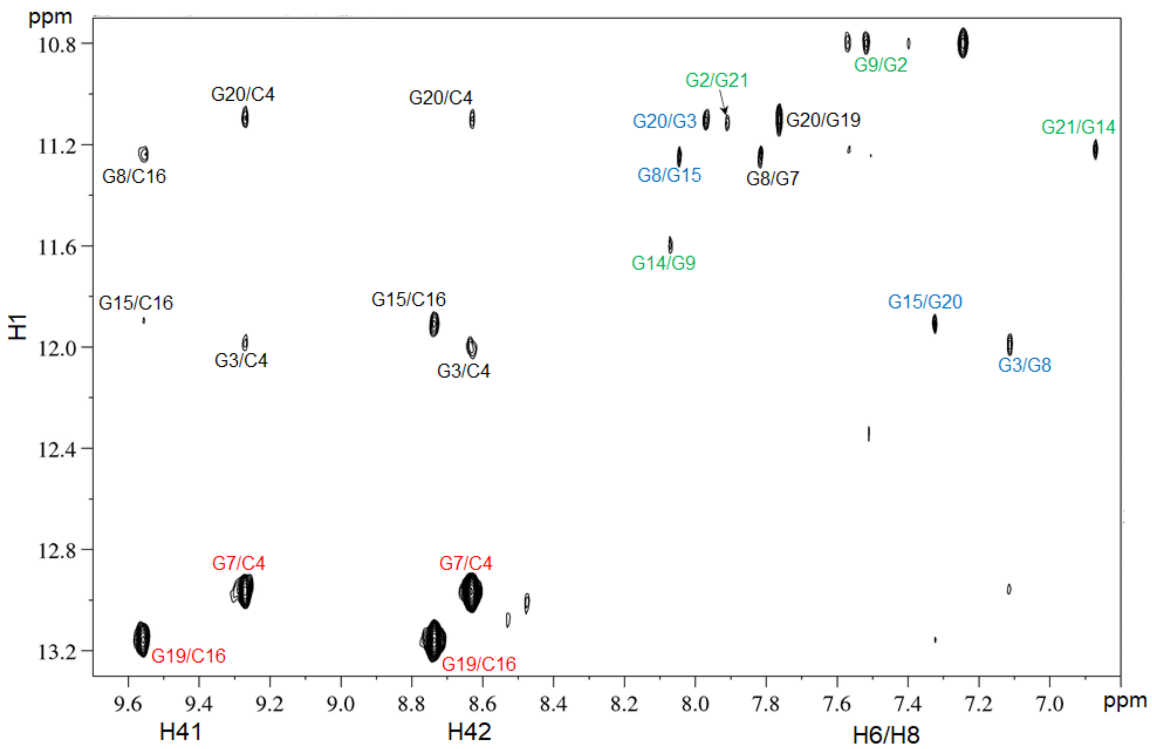

Figure S19. The NOESY H1-H41/H42/H6/H8 fingerprint region of *NOP56*-G4-PDS complex in 10% D_2O (mixing time = 200 ms). Internucleotide G H1-G H8 NOEs supporting the G2·G21·G14·G9 and G3·G8·G15·G20 G-tetrads are labeled in green and blue colors, respectively. Internucleotide G H1-C H41/H42 NOEs supporting the C4·G7·C16·G19 tetrad are labeled in red color. [DNA] = 0.5 mM, [PDS] = 1.5 mM, [NaPi, pH 7] = 1 mM, 25 °C.

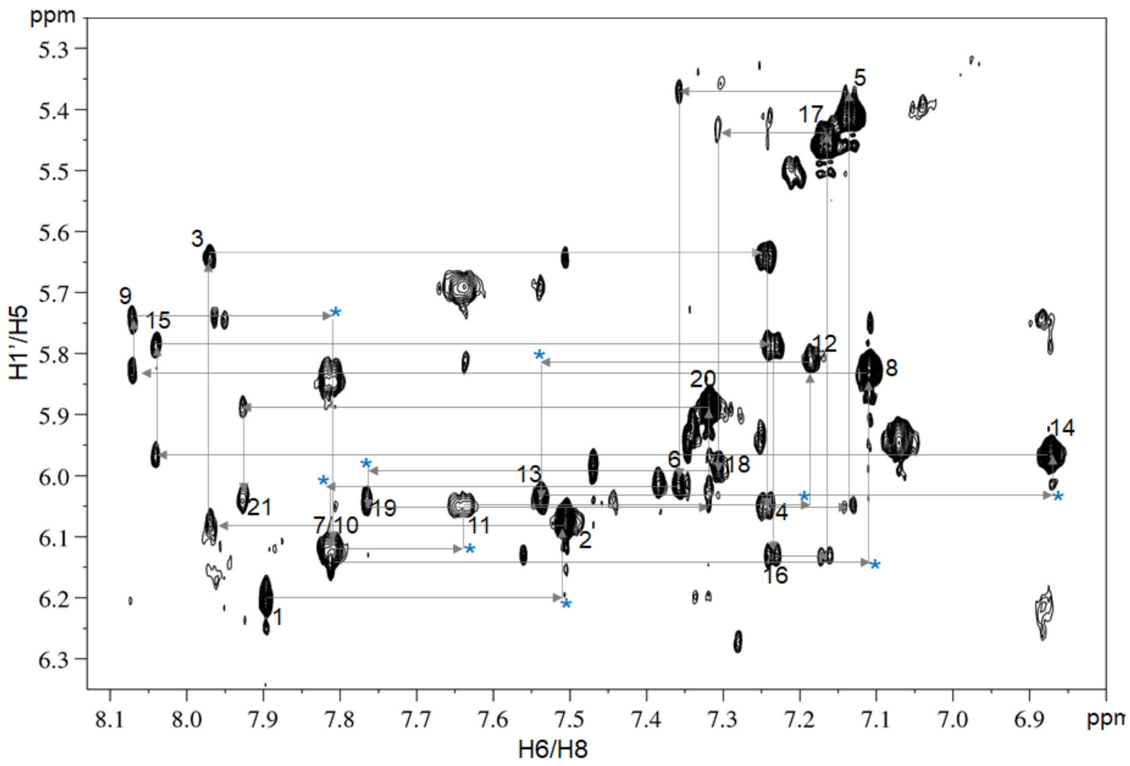

Figure S20. The NOESY H6/H8-H1′ region of *NOP56*-G4-PDS complex in 99.96% D_2O (mixing time = 300 ms). [DNA] = 0.5 mM, [PDS] = 1.5 mM, [NaPi, pH 7] = 1 mM, 25 °C. The missing sequential H8/H6-H1′ NOEs are indicated by blue asterisks.

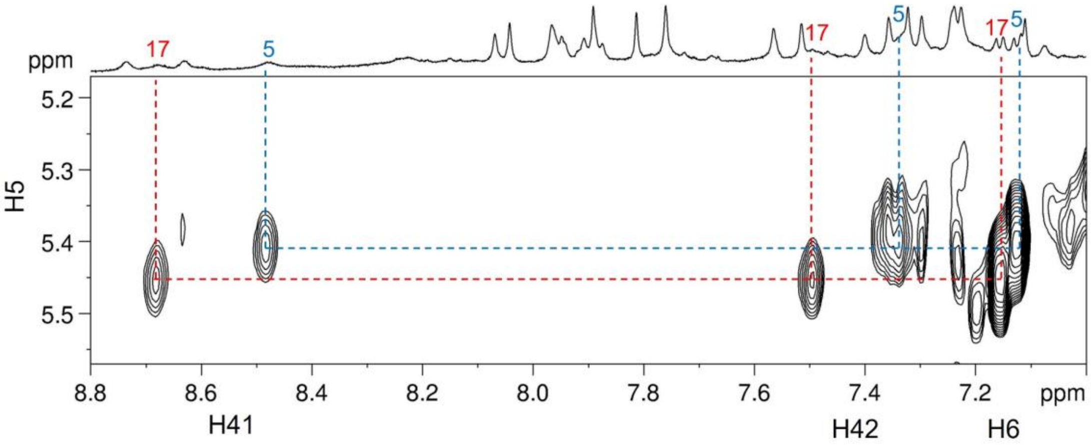

Figure 21. NOESY spectrum of *NOP56*-G4-PDS complex shows intranucleotide NOEs of C5 H5-H41/H42 (blue) and C17 H5-H41/H42 (red). [DNA] = 0.5 mM, [PDS] = 1.5 mM, [NaPi, pH 7] = 1 mM, 25 °C.

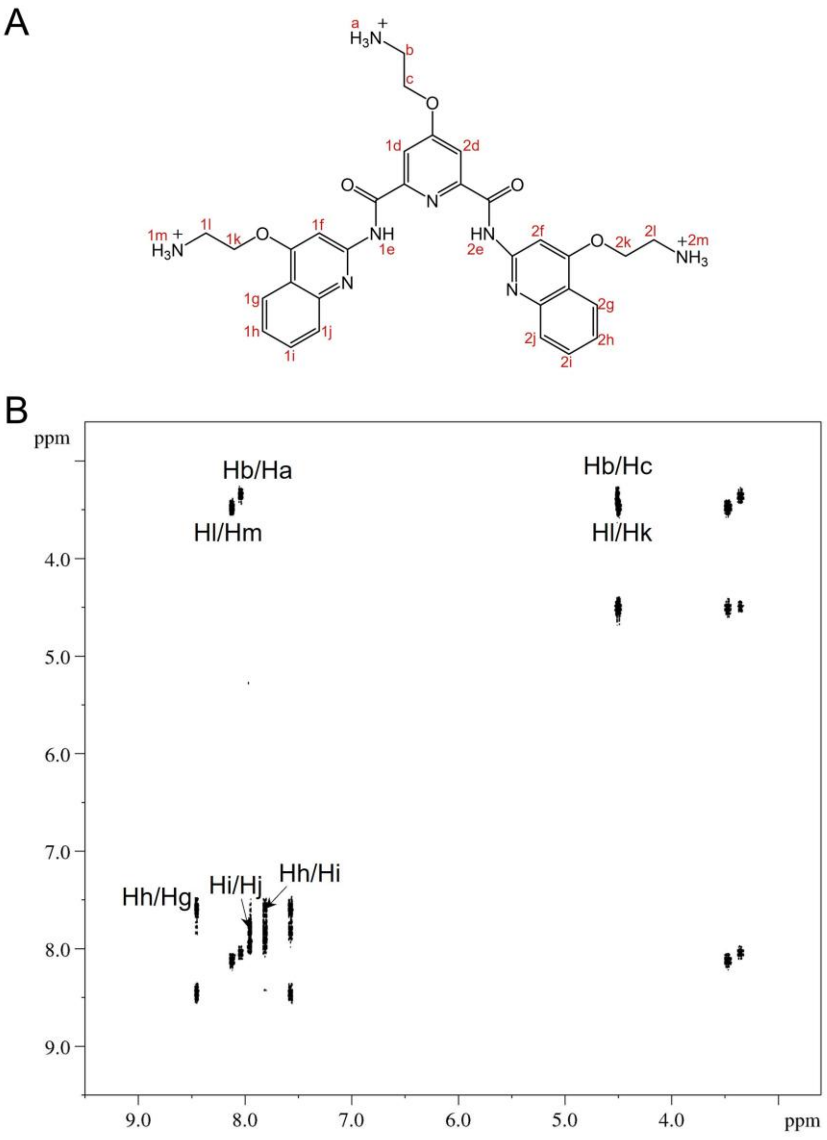

Figure S22. (A) Scheme of PDS chemical structure and (B) COSY spectrum of the free PDS. [PDS] = 1 mM, 99.9% DMSO-*d6*, 25 °C. The proton numbering of PDS is consistent with that reported in literature[4].

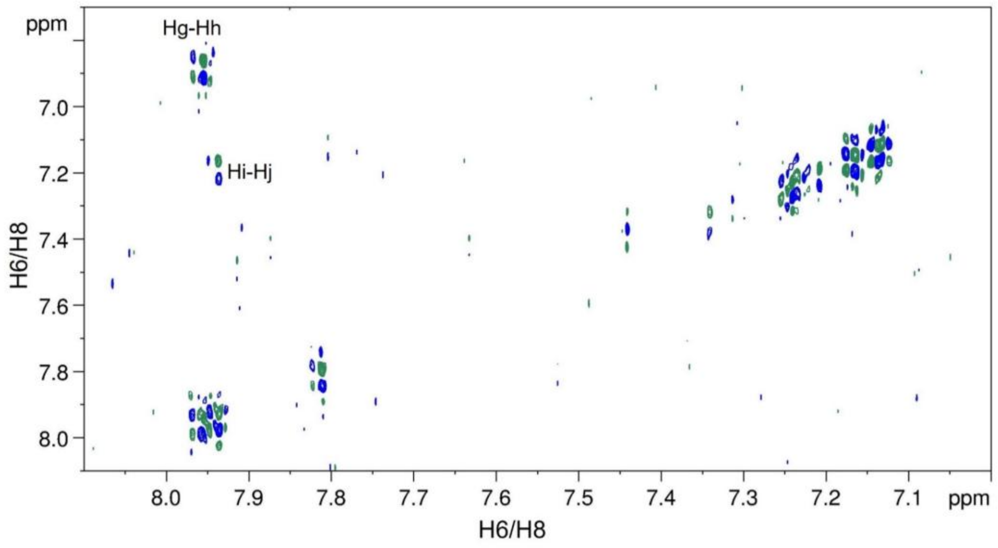

Figure S23. DQF-COSY spectrum shows assignment of PDS in the *NOP56*-G4-PDS complex. Phase-sensitive correlation peaks are shown in blue and green colors. [DNA] = 0.5 mM, [PDS] = 1.5 mM, [NaPi, pH 7] = 1 mM, 25 °C.

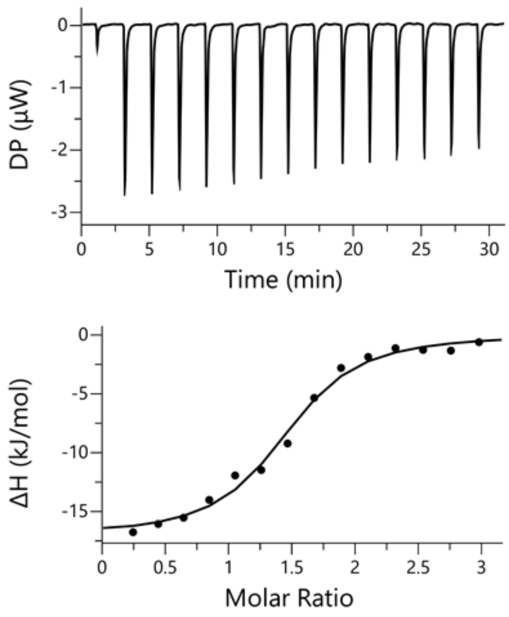

Experiment	1	2	3	Average	S.D.
K_d (μM)	0.63	0.66	0.62	0.64	0.02
n	1.41	1.27	1.28	1.32	0.08

Figure S24. ITC results of PDS binding to *NOP56*-G4. The binding stoichiometry (n) and dissociation constant (K_D) were determined to be 1.32 ± 0.08 and 0.64 ± 0.02 μM, respectively. Data are represented as mean ± S.D. by three replicative experiments.

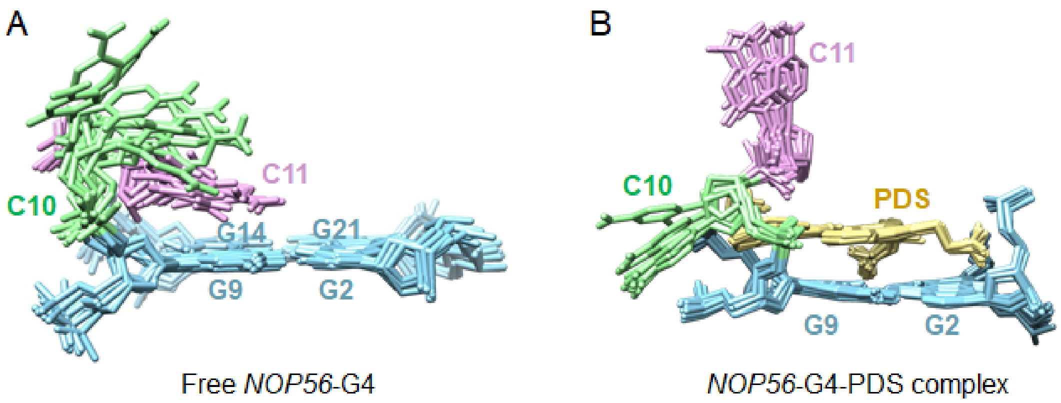

Figure S25. (A) C11 predominantly stacked on the terminal G2·G21·G14·G9 tetrad and C10 extruded out the G-core in the free *NOP56*-G4 structures (PDB ID: 8XGP, this work). (B) Both C10 and C11 extruded out the G-core due to binding of PDS at the terminal G2·G21·G14·G9 tetrad in the *NOP56*-G4-PDS complex structures (PDB ID: 9JX5, this work).

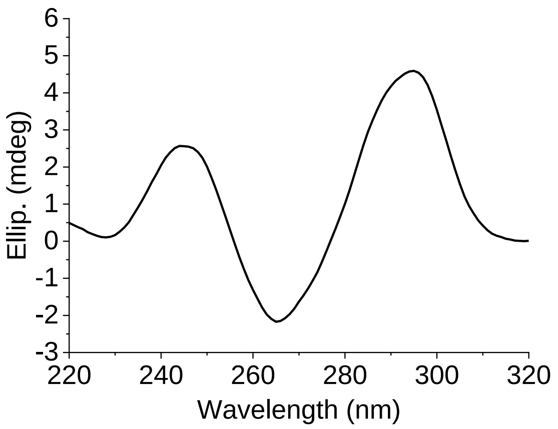

Figure S26. CD spectrum of *NOP56*-G4-PDS complex. [DNA] = 20 µM, [PDS] = 20 µM. [NaPi, pH 7] = 1 mM, [KCl] = 100 mM, 25 °C.

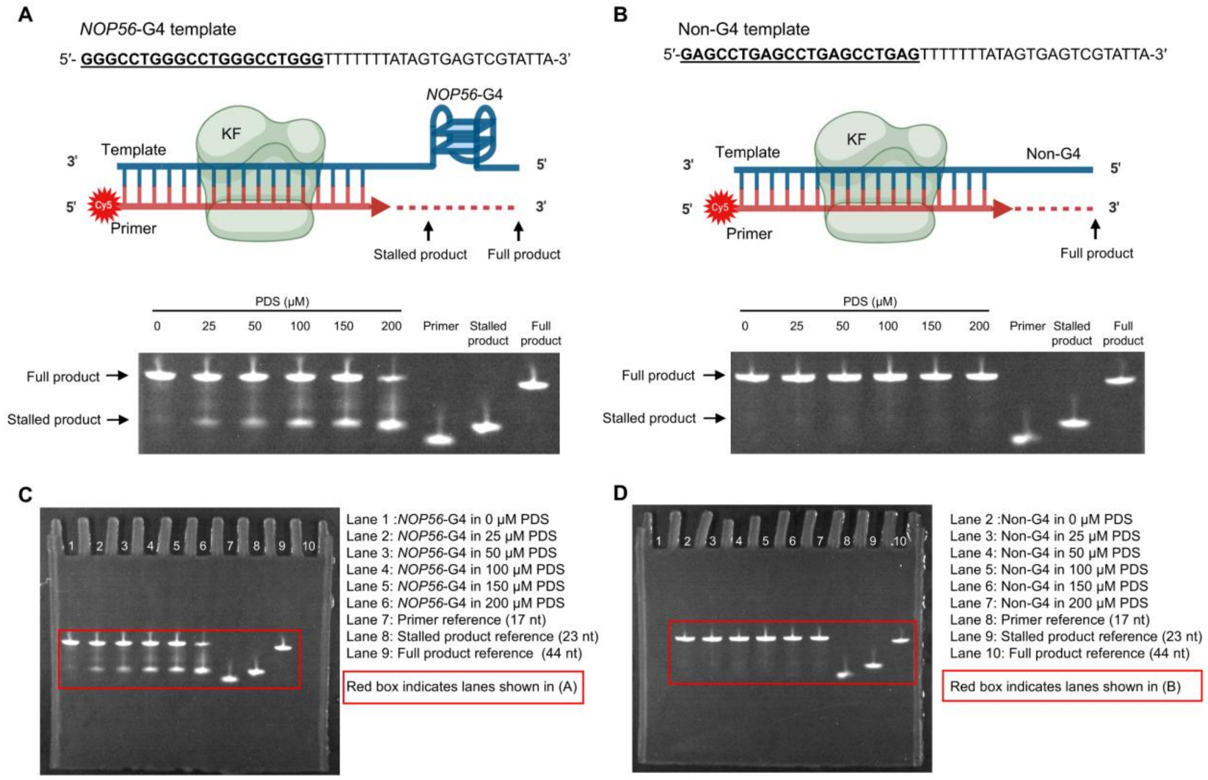

Figure S27. (A-B) Results of DNA polymerase stop assays using Klenow fragment (KF) on the *NOP56*-G4 template (A) and non-G4 template (B) under various PDS concentrations in a reaction buffer containing 100 mM KCl, 50 mM NaCl, 10 mM Tris-HCl (pH 7.9), 10 mM $MgCl_2$ and 1 mM DTT. The stalled products accumulated at higher PDS concentrations in the *NOP56*-G4 template system but not the non-G4 template system. (C-D) The original unmodified gel images for (A) and (B), respectively. The sequences of primer, primer reference, stalled product reference, and full product reference are shown in Table S1.

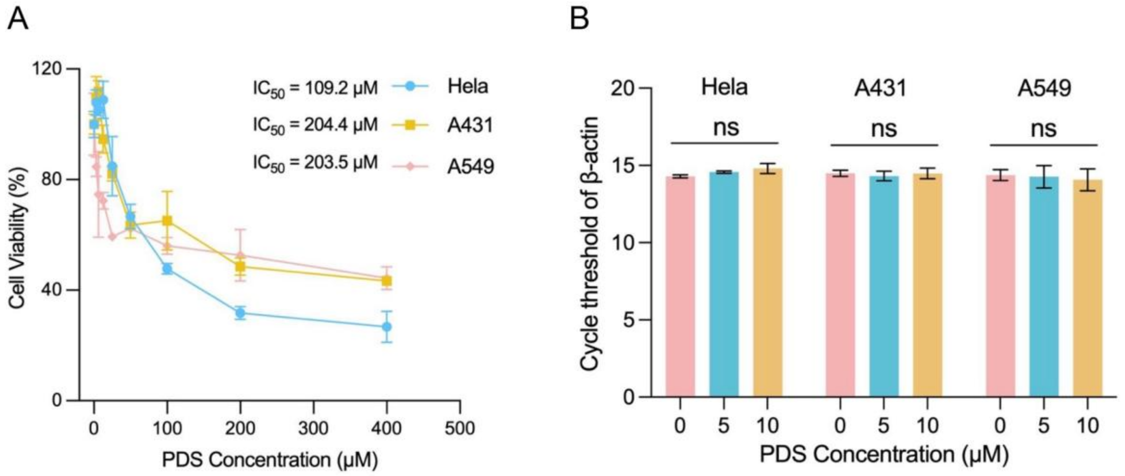

Figure S28. (A) Hela, A431 and A549 cell viability with the treatment of variable concentrations of PDS. Data are represented as mean ± SEM by three independent experiments. IC_{50} were calculated using log transformed to normalized distribution. (B) RT-qPCR results of *β-actin* mRNA levels in Hela, A431 and A549 cells after treatment with 0, 5 or 10 μM PDS for 24 h. Data are represented as mean ± SEM by three independent experiments. The statistical analysis was performed using one-way ANOVA followed by Dunnett post-hoc test. ns: not statistically significant.

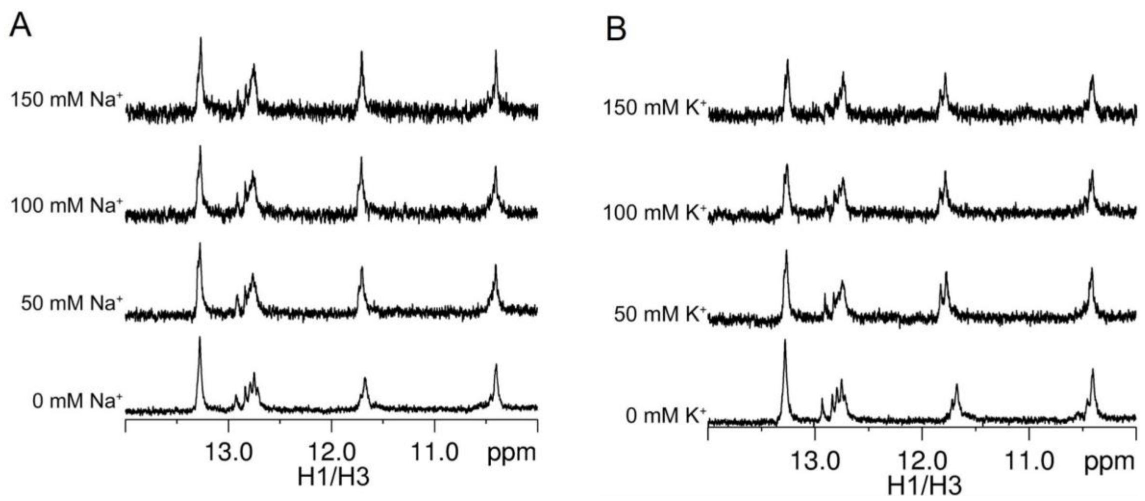

Figure S29. NMR spectra of the 21-nt RNA sequence r(GGGCCU)$_3$GGG in various concentrations of (A) NaCl and (B) KCl. The lack of multiple G H1 signals at 10.0 to 12.0 ppm suggested no G4 formation. The multiple G H1 signals at ~13.0 ppm arising from G-C Watson-Crick base pairs suggest that r(GGGCCU)$_3$GGG likely formed duplex and/or hairpin structures. [RNA] = 0.1 mM, [NaPi, pH 7] = 1 mM, 25 °C.

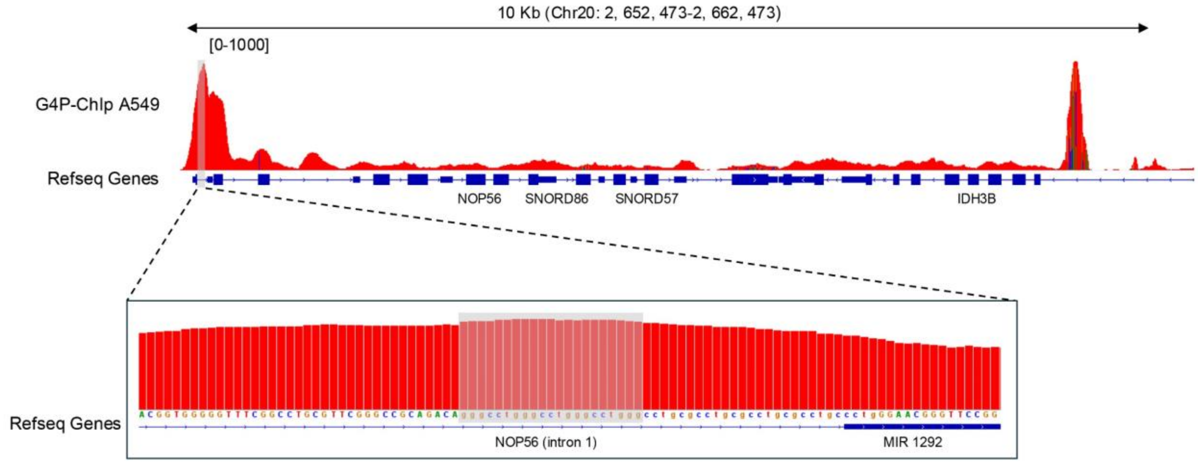

Figure S30. Genome browser view of the 10-Kb region based on the analysis of G4P-ChIP-seq data acquired in A549 cell line. The light grey shading highlights G4 peaks covering the (GGGCCT)$_3$GGG sequence in *NOP56* intron 1 (chr20: 2652733-2652753). The G4P-ChIP-seq data in A549 cell line was downloaded from the NCBI GEO datasets (GSE133379).[5] The G4P-ChIP-seq data was analyzed using established protocol[5]: (i) the original sequencing data in fastq format were aligned to the human genome (hg38) and generated the SAM file, (ii) the mapped reads were written to bam files after being filtered by the Samtools view and Samtools rmdup to removing low-quality alignments and PCR duplicates reads, and (iii) peaks of reads enrichment were identified using macs2[6] with the following parameters: –*q* value 0.001, –keep-dup 1, and default values for the other parameters.

References

[1] J. L. Markley, A. Bax, Y. Arata, C. W. Hilbers, R. Kaptein, B. D. Sykes, P. E. Wright, K. Wüthrich, *J. Biomol. NMR* **1998**, *12*, 1.
[2] J. N. Zadeh, C. D. Steenberg, J. S. Bois, B. R. Wolfe, M. B. Pierce, A. R. Khan, R. M. Dirks, N. A. Pierce, *J. Comput. Chem.* **2011**, *32* (1), 170.
[3] A. Kettani, S. Bouaziz, A. Gorin, H. Zhao, R. A. Jones, D. J. Patel, *J. Mol. Biol.* **1998**, *282* (3), 619.
[4] L. Y. Liu, T. Z. Ma, Y. L. Zeng, W. Liu, Z. W. Mao, *J Am Chem Soc* **2022**, *144* (26), 11878.
[5] K.W. Zheng, J.Y. Zhang, Y.D. He, J.Y. Gong, C.J. Wen, J.N. Chen, Y.H. Hao, Y. Zhao, Z. Tan, *Nucleic Acids Res.* **2020**, *48* (20), 11706.
[6] Y. Zhang, T. Liu, C. A. Meyer, J. Eeckhoute, D. S. Johnson, B. E. Bernstein, C. Nusbaum, R. M. Myers, M. Brown, W. Li, X. S. Liu, *Genome Biol.* **2008**, *9* (9), R137.
